# Genome-Wide Identification and Comparative Analysis of Cytosine-5 DNA Methyltransferase and Demethylase Families in Wild and Cultivated Peanut

**DOI:** 10.3389/fpls.2016.00007

**Published:** 2016-02-03

**Authors:** Pengfei Wang, Chao Gao, Xiaotong Bian, Shuzhen Zhao, Chuanzhi Zhao, Han Xia, Hui Song, Lei Hou, Shubo Wan, Xingjun Wang

**Affiliations:** Biotechnology Research Center, Shandong Academy of Agricultural Sciences, Shandong Provincial Key Laboratory of Crop Genetic Improvement, Ecology and PhysiologyJinan, China

**Keywords:** DNA mehtylation, C5-MTase, DNA demethylase, peanut, BAH domain, abiotic stress

## Abstract

DNA methylation plays important roles in genome protection, regulation of gene expression and is associated with plants development. Plant DNA methylation pattern was mediated by cytosine-5 DNA methyltransferase and demethylase. Although the genomes of AA and BB wild peanuts have been fully sequenced, these two gene families have not been studied. In this study we report the identification and analysis of putative cytosine-5 DNA methyltransferases (C5-MTases) and demethylases in AA and BB wild peanuts. Cytosine-5 DNA methyltransferases in AA and BB wild peanuts could be classified in MET, CMT, and DRM2 groups based on their domain organization. This result was supported by the gene and protein structural characteristics and phylogenetic analysis. We found that some wild peanut DRM2 members didn't contain UBA domain which was different from other plants such as Arabidopsis, maize and soybean. Five DNA demethylase encoding genes were found in AA genome and five in BB genome. The selective pressure analysis showed that wild peanut C5-MTase genes mainly underwent purifying selection but many positive selection sites can be detected. Conversely, DNA demethylase genes mainly underwent positive selection during evolution. Additionally, the expression dynamic of cytosine-5 DNA methyltransferase and demethylase genes in different cultivated peanut tissues were analyzed. Expression result showed that cold, heat or PEG stress could influence the expression level of C5-MTase and DNA demethylase genes in cultivated peanut. These results are useful for better understanding the complexity of these two gene families, and will facilitate epigenetic studies in peanut in the future.

## Introduction

DNA methylation is a process that DNA methyltransferase transfer the methyl group from the methyl donor S-adenosylmethionine (SAMe) to cytosine residues and generate S-adenosylhomocysteine (SAH) (Lu and Mato, 2012). DNA methylation is a conserved epigenetic gene regulation mechanism in plants and animals (Zhong et al., 2014). In a narrow sense, DNA methylation is DNA cytosine methylation which is catalyzed by cytosine-5 DNA methyltransferase through transferring the methyl group from S-adenosyl methionine into the 5′ position of the pyrimidine (Bender, 2004). DNA methylation is associated with gene silencing, X chromosome inactivation in females, and maintenance of genomic integrity in eukaryotes (Heard and Disteche, 2006; Klose and Bird, 2006; Feinberg, 2007). In animal, DNA methylation mainly occurred in CG sequence context, but in plants, it also occurred in other two sequence contexts: CHG and CHH (where H is A, C, or T) (Xiao et al., 2006; Rai et al., 2008; Hsieh et al., 2009).

Small RNAs could direct methylation through recruitment of methyltransferase. It is called RNA-directed DNA methylation (RdDM) pathway (Matzke and Mosher, 2014). At first, the small interfering RNAs (siRNA) were found to be a group of small RNAs that could direct the occurrence of DNA methylation. For example, the silence of many transposable elements (TEs) was due to siRNA-directed DNA methylation (Slotkin et al., 2009). Now, it is confirmed that microRNAs (miRNA) and piwi-interacting RNAs (piRNA) could also direct RdDM (Wu et al., 2010).

In eukaryotes, DNA methylation dynamic is controlled by three DNA methylation mechanisms including, *de novo* DNA methylation, DNA methylation maintenance, and DNA de-methylation (Law and Jacobsen, 2010). In plants, the key enzyme for *de novo* DNA methylation is DOMAINS REARRANGED METHYLTRANSFERASE 2 (DRM2). *De novo* DNA methylation in all sequence contexts depends on RdDM pathway. In animal, the homolog of DRM2 is DNA methyltransferase 3 (Dnmt 3; Law and Jacobsen, 2010; Zhong et al., 2014). Two key enzymes for DNA methylation maintenance are methyltransferase (MET) and chromomethylase (CMT; Du et al., 2012; Garg et al., 2014). The DNA methylation of CG sequence context is mainly maintained by MET (Henderson et al., 2010). In *Arabidopsis*, the *met1* mutation and MET1 antisense gene transgenic lines, displayed genome wide hypomethylation to compare with the wild type (Kim et al., 2008). CMT mainly maintains the methylation of CHH and CHG sequence contexts (Noy-Malka et al., 2014). The CMT was plant-specific methyltransferase. In animal, the homology of MET is DNA methyltransferase 1 (Dnmt1). In *Arabidopsis*, three CMT genes were found, namely *AtCMT1, AtCMT2*, and *AtCMT3* (Garg et al., 2014). However, in moss *Physcomitrella patens*, only *PpC*MT, a homolog of *AtCMT3*, was identified (Noy-Malka et al., 2014).

All C5-MTases contain methyltransferase domain, but proteins contained methyltransferase domain are not necessary C5-MTases. DNMT2 type proteins which represent the transfer tRNA MTase (Garg et al., 2014) which were not included in our study.

Generally, level of DNA methylation is determined mainly by the common action of both C5-MTases and demethylases (Cao et al., 2014). In *Arabidopsis*, four demethylases family members were found, including repressor of silencing 1(ROS1), demeter (DME), demeter-like2 (DML2), and demeter-like3 (DML3). Mutation of these genes resulted in increased level of DNA methylation in all sequence contexts at specific genomic loci comparing with the wild type lines (Penterman et al., 2007). These four demethylases were also DNA glycosylase/lyase. ROS1, DME, DML2, and DML3 can excise 5-meC from all sequence contexts (Penterman et al., 2007; Zhu, 2009). Studies showed that ROS1 gene, a 5-meC DNA glycosylase/demethylase, active DNA de-methylation in extensive tissues and cells. But DME gene preferentially expressed in central cell and was more associated with allelic-specific DNA de-methylation (Zhu, 2009).

Peanut is an important crop plant for human nutrition and grown world widely. Peanut is allotetraploid (AABB, 4*n* = 4*x* = 40) originated from a single hybridization event between AA and BB wild species, and subsequently underwent spontaneous genome duplication (Kochert et al., 1996; Freitas et al., 2007; Moretzsohn et al., 2013). Recently, whole genome sequencing of the two progenitor wild peanut is complete, the assembled AA and BB genome sequences are available (http://peanutbase.org/). In the present study, we have identified and analyzed C5-MTases and demethylases from AA genome and BB genome.

The phylogenetic relationship among C5-MTases and demethylases in wild peanut has been analyzed. In addition, three-dimensional (3D) structure modeling of selected members was carried out to understand their function. The allotetraploid genome sequencing data and RNA-seq data showed that these C5-MTases and demethylases found in wild peanut could also be found in cultivated peanut. To understand the function of these genes in cultivated peanut, we carried out the gene expression analyses of cultivated C5-MTase and demethylase encoding genes in various tissues/developmental stages and stress conditions. Our analyses provide the framework for future functional studies of these important gene families in peanut.

## Materials and methods

### Data collection and identification of C5-MTases and DNA demethylases

Amino acid sequences of all *Arabidopsis thaliana* C5-MTases and DNA demethylases were collected from the National Center for Biotechnology Information (NCBI) database (http://www.ncbi.nlm.nih.gov/). All protein sequences of *A. thaliana* C5-MTases and DNA demethylases were used to search the hidden Markov model (HMM) of the conserved key domain of C5-MTases and DNA demethylases using the Pfam online software (version 27.0) (http://pfam.xfam.org/). Gene ID of all *A. thaliana* C5-MTases and DNA demethylases were showed in Table S1.

The conserved key domain of C5-MTase is the DNA methylase domain and the HMM ID of this domain is PF00145 in pfam database. The conserved key domain of DNA demethylases is the HhH-GPD domain and the HMM ID of this domain is PF00730 in pfam database. Additionally, majority of DNA demethylases contained RRM_DME domain and the HMM ID of this domain is PF15628 in pfam database. We employed the amino acid sequences of two HMMs as queries to identify all possible C5-MTase and DNA demethylase protein sequences in the AA and BB genome database (http://peanutbase.org/) genomes using the BLASTP program (*E* < 0.001). SMART online software (http://smart.embl-heidelberg.de/) and domain search tool of Pfam were used to confirm and classify each putative peanut C5-MTase or demethylase. Wild Peanut C5-MTases or DNA demethylases were named based on the best hit proteins by NCBI-blastp.

The basic information of C5-MTase and DNA demethylase genes in wild peanut were generated from peanut genome database (http://peanutbase.org/). Protein isoelectric point (pI) and molecular weight (Mw) of C5-MTases and DNA demethylases in peanut were analyzed using online Expasy software (http://web.expasy.org/compute_pi/).

The latest version of genomes, proteins, and cDNA sequences of other species were collected from plant ensemble database (http://plants.ensembl.org/index.html). The respective genome database are as follows: *Arabidopsis thaliana* genome database (http://www.plantgdb.org/AtGDB/), *Glycine max* genome database (http://www.plantgdb.org/GmGDB/), *Zea may* genome database (http://www.plantgdb.org/ZmGDB/), *Solanum lycopersicum* genome database (http://www.plantgdb.org/SlGDB/), *Oryza sativa* genome database (http://www.plantgdb.org/OsGDB/), *Lotus japonicus* genome database (http://www.plantgdb.org/LjGDB/), *Medicago truncatula* genome database (http://www.plantgdb.org/MtGDB/), *Cajanus cajan* genome database (http://gigadb.org/dataset/100028), *Cicer arietinum* genome database (http://nipgr.res.in/CGAP/home.php) and *Physcomitrella patens* genome database (http://www.cosmoss.org/).

### Chromosomal location and gene structure of wild peanut C5-MTase and DNA demethylase genes

The chromosomal location of wild peanut C5-MTases and DNA demethylase genes was obtained from peanut genome database. The chromosomal location map was plotted using Circos software (Krzywinski et al., 2009). The gene structures of the wild peanut DNA C5-MTase and demethylase genes were plotted using online software Gene Structure Display Server 2.0 (http://gsds.cbi.pku.edu.cn/) (Guo et al., 2007).

### Multiple sequence alignments and phylogenetic analysis

Multiple sequence alignments using ClustalW2 online software (http://www.ebi.ac.uk/Tools/msa/clustalw2/) were performed using the full-length protein sequences of wild peanut C5-MTase and DNA demethylase. Neighbor-Joining (NJ) and Maximum likelihood (ML) trees were constructed using MEGA 4.0 with the aligned protein sequences (Tamura et al., 2007). To support the calculated relationship, 1000 bootstrap samples were generated.

In order to analyze the classes and subgroups of the peanut C5-MTases and DNA demethylase families, *P. patens* MET (UniProtKB ID: A9RVR7), and CMT (UniProtKB ID: A9ST18; Malik et al., 2012), 10 *A. thaliana* C5-MTases, 4 *A. thaliana* DNA demethylases, 7 *S. lycopersicum* C5-MTases, and 3 *S. lycopersicum* DNA demethylases (Cao et al., 2014), 7 *Z. may* C5-MTases (Candaele et al., 2014), 8 *G. max* C5-MTases, 8 *L. japonicus* C5-MTases, 6 *O. sativa* C5-MTases, 5 *C. cajan* C5-MTases, 6 *C. arietinum* C5-MTases, and 7 *M. truncatula* C5-MTases (Garg et al., 2014) (gene ID of all C5-MTases and DNA demethylases were showed in Table S1) were included in the phylogenetic analysis by generating a NJ tree (Poisson correction, pairwise deletion, and bootstrap = 1000 replicates).

### Conserved motif analysis of C5-MTases and DNA demethylases in wild peanut

Full length amino acid sequences of peanut C5-MTases and DNA demethylases were used by the MEME tool (Bailey et al., 2006) (http://meme.nbcr.net/meme/) to identify conserved motifs (Parameter setting: output motifs: 20; minimum motif width: 6; maximum motif width: 200). The conserved motif sequence information of wild peanut C5-MTase and DNA demethylase was listed in Table S2.

### Homology modeling

The templates for homology modeling were selected using the best hit in BLAST searches against the PDB database. Homology model of the protein was generated using SWISS-MODLE online software (http://www.swissmodel.expasy.org/interactive). At least 186 models for each protein were generated using “building model” engine and the best model was selected based on the best global model quality estimation (GMQE). QMEAN4 scoring was used to evaluate and check for the possible errors in homology model of the proteins.

### Molecular dynamic simulations of protein domain

Molecular dynamics simulations were performed for 9 ns using the GROMACS package v. 4.5.3 (Van Der Spoel et al., 2005) and trajectories for protein domain in aqueous solution were analyzed to obtain structural and dynamic properties using the GROMACS analysis tools package. The interaction potential energy between the monomers of the dimers, root mean square deviation (RMSD), solvent accessible surface area (SASA), and collective motions (essential dynamics using g_covar and g_anaeig analysis in the GROMACS package) of the protein domain backbone during the simulation time.

### Plant material, stress treatments, and RNA isolation

Peanut (*Arachis hypogaea* L.) cv. Luhua-14 was used in this experiment. Plant tissues including roots, stems, leaves, flowers, seeds, and 6 stages of gynophores [gynophore tip containing embryo; Stage 1 gynophores (S1): aerial grown with green or purple color; Stage 2 gynophores (S2): three day after soil penetration; Stage 3 gynophores (S3): nine days after soil penetration (Xia et al., 2013); Stage 4 gynophores (S4): 12 days after soil penetration; Stage 5 gynophores/pods (S5): 16 days after soil penetration; Stage 6 gynophores/pods (S6): 20 days after soil penetration] were used for gene expression analysis. All samples were cleaned quickly and immediately frozen in liquid nitrogen before stored at –80°C for RNA extraction and gene expression analysis. Three biological replicates of all tissues were prepared.

Twelve-day-old peanut seedlings were subjected to various abiotic stresses including 20% PEG 6000, cold (8°C) and heat treatment (42°C) treatments according to previously described methods (Chen et al., 2006). Leaf samples were collected at 0, 3, 6, 12, 24, and 48 h after treatment and immediately frozen in liquid nitrogen. Three independent biological replicates were used in this experiment.

Samples were incubated with 500 μl CTAB at 65°C for 15 min. Two hundred and fifty microliters of chloroform and water saturated phenol was added and mixed thoroughly. After centrifugation at 12,000 g for 10 min at 4°C, the supernatant was collected and equal volume of chloroform was added and mixed by vortex. After centrifugation at 12,000 g for 10 min at 4°C, the aqueous phase which containing the RNA was transferred to a clean tube and was precipitated with equal volume of isopropyl alcohol and freeze for 8 h at −20°C. RNA was precipitated by centrifugation at 12,000 g for 20 min at 4°C. RNA was washed with 75% ethanol, air dried, and re-suspended with RNAase-free H_2_O. For reverse transcription, the first-strand cDNA was synthesized with an oligo (dT) primer using a PrimeScript™ first-strand cDNA synthesis kit (TaKaRa).

### Gene expression analysis

qRT-PCR was carried out using FastStart Universal SYBR Green Master (ROX) with a 7500 real-time PCR machine (ABI). The following PCR program was used: 95°C for 30 s, 40 cycles (95°C for 5 s, 60°C for 30 s). Data were quantified using the ^2−ΔΔ^Ct method based on Ct values of peanut C5-MTases and DNA demethylase genes and β-actin. The primers for qRT-PCR were provided in Table S3. Heat map of was generated based on the RPKM values using the Multiexperiment View software. PRKM values were based on transcriptome data (data not shown).

### Promoter cis-acting regulatory element analysis

Promoters of wild peanut DNA C5-MTase and demethylase encoding genes were analyzed with the online software Plantcare (http://bioinformatics.psb.ugent.be/webtools/plantcare/html/) to find the cis-acting regulatory element.

### NLS predicted and sub-cellular localization

The nuclear localization signal in the proteins was analyzed using the online software cNLS Mapper (http://nls-mapper.iab.keio.ac.jp/cgi-bin/NLS_Mapper_form.cgi). The full-length coding sequences (CDs) of C5-MTase and DNA demethylase genes were amplified by reverse transcription PCR using gene-specific primers and cloned in pMD18-T plasmid (Takara). The CDs sequence of DRM2, CMT2, CMT3, and ROS1 were fused in-frame, upstream of GFP in pBI221-GFP vector. The primers for PCR were provided in Table S4.

The recombinant vectors and the pBI221-GFP control vector were transformed into onion epidermal cells, respectively, using the particle delivery system (Bio-Rad Biolistic PDS-1000/He). The transformed onion epidermal cells were cultured on Murashige and Skoog (MS) plates at 25°C for 24 h in dark. Subsequently, nuclei were stained with 1 μg/mL of 4′, 6-diamidino-2-phenylindole (DAPI) in phosphate-buffered saline for 10 min at room temperature (Sharma et al., 2013). The cultured transformed onion epidermal cells were visualized using a fluorescence microscope (Olympus Microsystems).

### Syntenic and selective pressure analysis

The analysis of synteny between AA and BB genomes was based on comparisons of the blocks of 100 kb chromosome containing C5-MTase or DNA demethylase genes. All wild peanut C5-MTase and DNA demethylase genes were set as the anchor points according to their chromosome locations. A syntenic block is defined as a region in which three or more conserved homology genes were presented. The homology gene pairs from blocks were identified by local all-vs.-all BLASTN (*E* < 10^−20^) (Sato et al., 2008; Lin et al., 2014).

The Ka, Ks and ω rates (Ka/Ks ratios) between gene pairs or gene families were calculated using the codeml program under PAML (phylogenetic analysis maximum likelihood) V. 4.7 software (Yang, 2007). This program contained six site models: M0, M1a (nearly neutral), M2a (positive selection), M3 (discrete), M7 (beta), and M8 (beta and ω). M0 and M3 comparison can be used to test ω values vary among sites. M0-M3, M1a-M2a, and M7-M8 comparisons can be used to find positive selection sites (Yang et al., 2000). The Bayes Empirical Bayes (BEB) was used to calculate the Bayesian posterior probability (BPP) of the codon sites under a positive selection (Yang et al., 2005).

## Results

### Classification of C5-MTase and DNA demethylase genes in wild peanut

In our study, 14 C5-MTase coding genes were identified in wild peanut. The information of these peanut C5-MTase family members was showed in Table 1. Seven C5-MTase coding genes were discovered from AA genome and seven from BB genome. All domains and their positions in these protein sequences were list in Table S5. In wild peanut, C5-MTase gene length varied from 1740 to 4668 bp and protein length varied from 580 to 1556 aa.

**Table 1:** **C5-MTase and demethylase genes identified in wild peanut**.

**Gene name**	**Chromosome location**	**Gene ID**	**ORF length (bp)**	**No. of exons**	**Protein length (aa)**
MET1-A	A05:82542342.82549984	Aradu.GN4F8	4668	12	1556
MET1-B	B05:144815235.144822842	Araip.RYZ61	4668	12	1556
DRM2-A	A05:86867193.86874459	Aradu.NFN5F	1809	9	603
DRM2-B	B05:141574185.141584809	Araip.B5M84	1821	9	607
DRM2X1-A	A01:94847635.94853351	Aradu.DS6B6	2103	9	701
DRM2X1-B	B01:132495695.132501377	Araip.IV2RH	2112	9	704
DRM2X2-A	A05:23654289.23658131	Aradu.0Q9GS	1740	8	580
DRM2X2-B	B05:24020296.24024084	Araip.YNX4D	1740	8	580
CMT1-A	A05:3796815.3808030	Aradu.2P9P8	2466	19	822
CMT1-B	B05:3729600.3739701	Araip.V0N49	2460	19	820
CMT2-A	A01:12324484.12335096	Aradu.6W0PK	3222	23	1074
CMT2-B	B10:179655.190994	Araip.71XM6	3285	23	1095
CMT3-A	A05:89596488.89605805	Aradu.34YIE	2529	21	843
CMT3-B	B05:139158887.139168232	Araip.527SE	2571	21	857
ROS1-A	A02:93077515.93085966	Aradu.46ZNY	4515	20	1505
ROS1-B	B02:107822145.107830922	Araip.2A0J4	4752	20	1584
DME-A	A09:106368426.106380447	Aradu.GHV73	5160	20	1720
DME-B	B09:129890609.129902670	Araip.81XFD	5385	20	1795
DMElike-A	A08:27708497.27730088	Aradu.4D5YM	3186	19	1062
DMElike-B	B08:4956310.4963917	Araip.Z24EK	2151	19	717
ROS1X2-A	A06:63337260.63347864	Aradu.58JFQ	4722	18	1574
ROS1X2-B	B06:74890020.74902183	Araip.71PVU	6180	18	2060
ROS1like-A	A01:72619617.72624721	Aradu.2PP17	903	4	301
ROS1like-B	B01:102499036.102501657	Araip.QZ6BX	912	4	304

From moss (*P. patens*) to leguminous (*C. cajan, C. arietinum, G. max, M. truncatula*, and *L. japonicus*) or other seed plants (*A. thaliana, S. lycopersicum, O. sativa*, and *Z. may*), C5-MTases can be divided into three groups (MET, CMT, and DRM2) based on domains located in the N-terminal region of DNA methylase domain in these C5-MTases. Wild peanut C5-MTase names were assigned based on names of best hit proteins by NCBI-blastp. MET group members contained one replication foci domain (RFD) and two bromo adjacent homology (BAH) domains. CMT group members contained one chromo domain (Chr) which located in DNA methylase domain, and one bromo adjacent homology (BAH) domain. DRM2 group members contained ubiquitin associated (UBA) domain in some species (Malik et al., 2012; Candaele et al., 2014; Cao et al., 2014; Garg et al., 2014). Wild peanut C5-MTases also can be divided into three groups based on the domains located in the N-terminal region of DNA methylase domain. AA genome wild peanut MET and CMT group included the MET-A, CMT1-A, CMT2-A, and CMT3-A. BB genome wild peanut MET and CMT group included the MET-B CMT1-B, CMT2-B, and CMT3-B.

AA genome wild peanut DRM2 group included DRM2-A, DRM2X1-A, and DRM2X2-A. BB genome wild peanut DRM2 group included DRM2-B, DRM2X1-B, and DRM2X2-B. Interestingly, not all wild peanut DRM2 members contained UBA domain (Table S5). Both DMR2-A and DRM2-B contained an extra incomplete DNA methylase domain. *MET-A, CMT1-A, CMT2-A, CMT3-A, DRM2-A, DRM2X1-A*, and *DRM2X2-A* in AA genome, respectively, were orthologous of *MET-B, CMT1-B, CMT2-B, CMT3-B, DRM2-B, DRM2X1-B*, and *DRM2X2-B* in BB genome. These C5-MTase gene sequences were extremely similar to their orthologous (Table S6). For example, MET-A protein and coding gene displayed 99% identity with MET-B protein and coding gene. This result reflected the high level of similarity between peanut AA and BB genomes. DRM2-A protein and coding gene displayed 99% identity with DRM2-B protein and coding gene. Other C5-Tases between these two diploid progenitors also showed high identity with their orthologous.

Ten DNA demethylases were identified in peanut, five from AA genome and five from BB genome. The information of these peanut DNA demethylase family members was showed in Table 1. All domains in these DNA demethylase sequences were listed in Table S5. All these DNA demethylase contained conserved HhH-GPD domain (Figure S1). ROS1-like-A and ROS1-like-B didn't contain RRM_DME domain. Some of these proteins contained Perm-CXXC domain (Table S5). The length of wild peanut DNA demethylase genes varied from 912 to 6180 bp, while the protein length varied from 301 to 2060 aa. Based on names of the best-hit proteins by NCBI-blastp, these 10 demethylases were named ROS1-A, ROS1-like-A, ROS1X2-A, DME-A, DME-like-A, ROS1-B, ROS1-like-B, ROS1X2-B, DME-B, and DME-like-B. ROS1-A, ROS1-like-A, ROS1X2-A, DME-A, and DME-like-A were identified in AA genome, while the orthologus ROS1-B, ROS1-like-B, ROS1X2-B, DME-B, and DME-like-B were identified in BB genome. These DNA demethylase genes and proteins were highly conserved, for example, the gene and protein sequences of ROS1-A displayed 96% identity with ROS1-B (Table S6).

**Figure 1:**
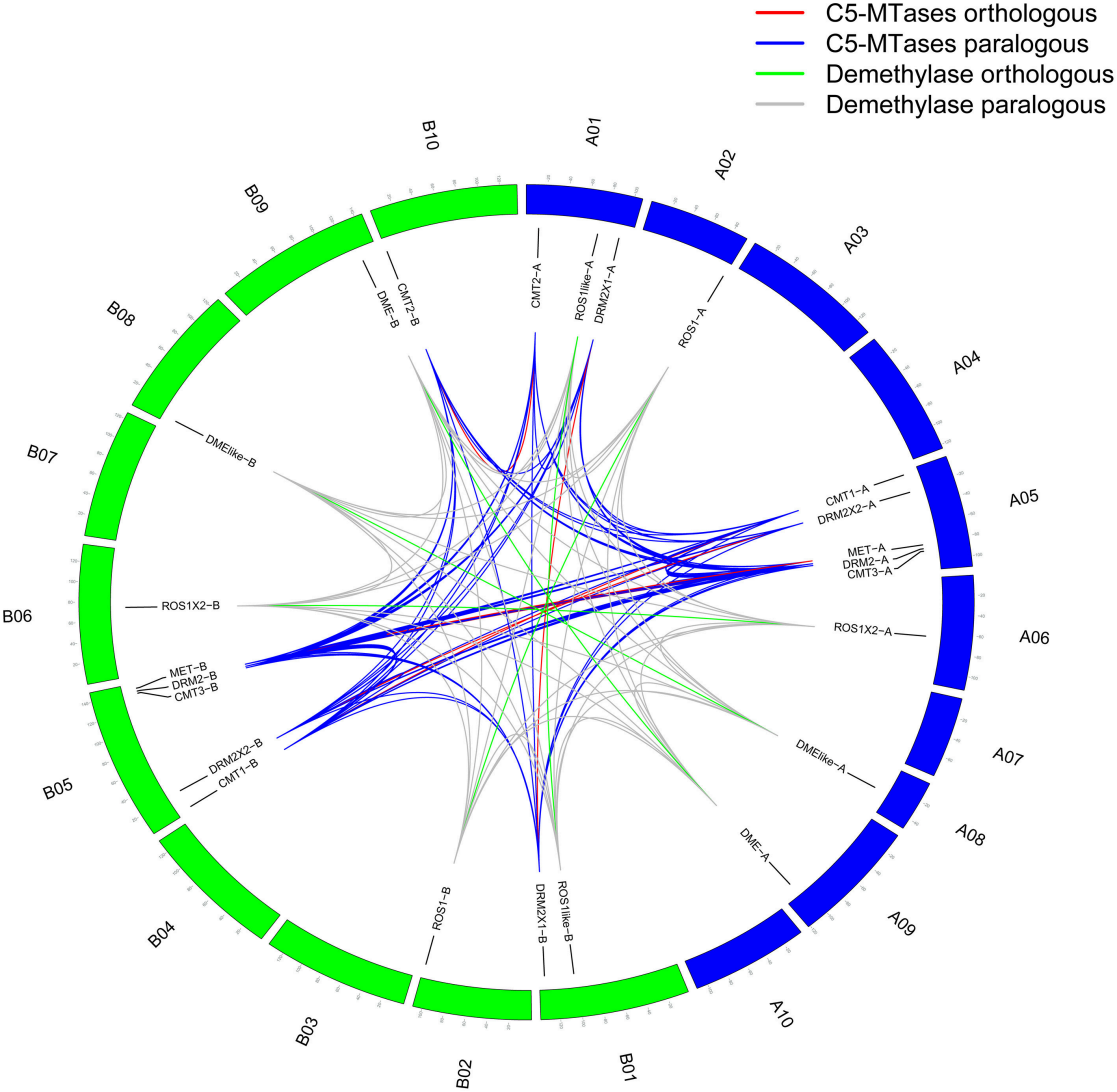
**Genome location of peanut C5-MTase and demethylase genes**. Blue represents AA genome chromosomes and green represents BB genome chromosomes.

Five C5-MTase genes from AA genome are distributed on A05 chromosome and others are distributed on A01 chromosome. Similarly five C5-MTase genes from BB genome are distributed on B05 chromosome, one is on B10 chromsome and others are on B01 chromosome. Most of the orthologous genes we identified are located on similar chromosome loci, for example CMT1-A (A05:3796815-3808030) and CMT1-B (B05:3729600-3739701) (Figure 1 and Table 1).

### Sequence analysis of wild peanut C5-MTase and DNA demethylase

We analyzed the protein and gene sequences of wild peanut C5-MTase and DNA demethylase. We predicted and verified motifs in C5-MTases and DNA demethylases of wild peanut and some other species including moss, monocots, dicots, and legumes. Totally, 20 conserved motifs (*E* ≤ 2.3) were found in C5-MTase family proteins. All C5-MTase members contained motif 5. Both MET group and CMT group members contained motif 1, 2, and 15. Only DRM2 group contained motif 4, 6, and 13. Based on motif type, proteins from same group were conserved, except PpCMT which contained only motif 5 and 11 (Figure S2A).

In DNA demethylase family, 20 conserved motifs (*E* ≤ 6.1e^−23^) were found. All DNA demethylases contained motif 5 and 14. Most DNA demethylases contained motif 1, 9, 16, and 17. Only DME-A and DME-B contained motif 8, 11, 13, and 18. ROS1-like-A and ROS1-like-B contained fewer conserved motifs that were different from other members. Each DNA demethylase was similar to its orthologous base on motif type and distribution (Figure S2B).

The methyltransferase domain of C5-MTases contained several conserved motifs (I, IV, VI, VIII, IX, and X) that play important roles for transferring methyl group from SAMe onto cytosine (Malone et al., 1995). In other legumes, the conserved sequences of these motifs have been identified (Garg et al., 2014). In wild peanut, we found that all C5-MTase family members contained these six motifs. The distribution order of motifs in wild peanut MET and DRM2 group members was different. But in peanut CMT group, the distribution order of motifs was the same as in MET group proteins. The motif distribution order in wild peanut C5-MTases was similar to other legumes (Figure S3).

**Figure 2:**
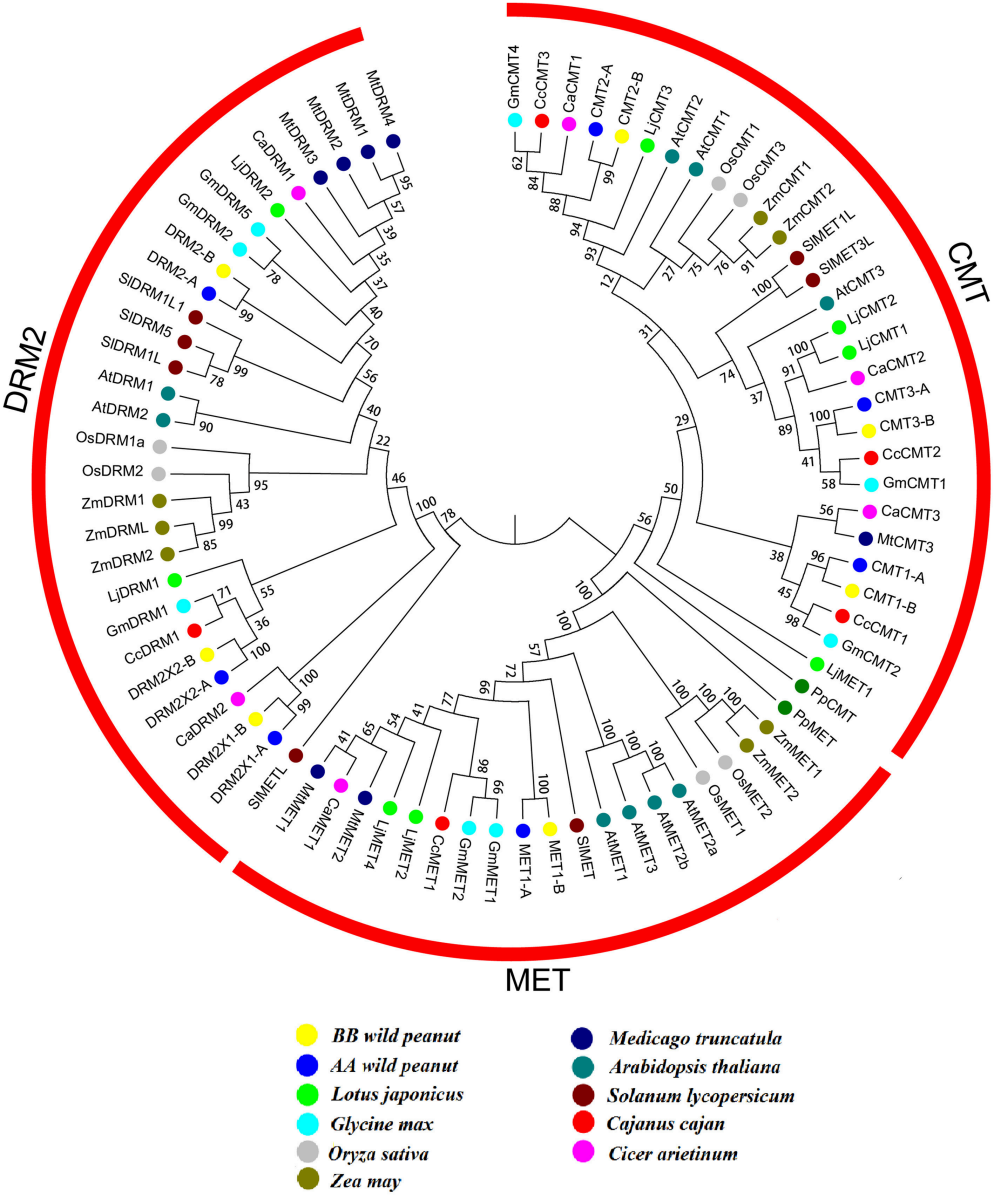
**Neighbor-joining phylogeny of C5-MTases**. Different species were distinguished by different colors.

Full-length protein sequences of C5-MTase and DNA demethylase from wild peanut and other 10 species (*C. cajan, C. arietinum, P. patens, O. sativa, Z. may, A. thaliana, S. lycopersicum, G. max, Lotus japonicas*, and *M. truncatula*) were used for construction of phylogenetic tree. The phylogenetic tree showed that C5-MTase family could be divided into MET, CMT, DRM2 groups. The evolution relationship between MET and CMT group was more close based on phylogenetic analysis. All members of MET and CMT group contained BAH domain. MET group could be divided into four subgroups, the legumes, dicots, monocots and moss subgroups. But in CMT or DRM2 group, the evolution relationship between these group members was complex (Figure 2).

Wild peanut DNA demethylase could be divided into two groups. ROS1-like-A and ROS1-like-B were clustered into a single group showing that these two members were quite different from other members. The ROS1-A, ROS1-B, ROS1X2-A, and ROS1X2-B were clustered together (Figure S4). The exon-intron structures of C5-MTases and DNA demethylases supported their phylogenetic analysis result. In C5-MTases, exon-intron structure of members in one group or subgroup was similar to members in other group or subgroup. In DNA demethylases, the exon-intron structures of ROS1-like-A and ROS1-like-B were different from other DNA demethylase family members (Figure S5).

Promoters of all wild peanut C5-MTase and DNA demethylase genes (2000 bp upstream of CDs) were analyzed. Table S7 showed the cis-elements of these promoters associated with abiotic stresses. Most promoters of orthologous genes contained similar cis-elements, and only a small number of orthologous gene promoters contained different cis-elements. The promoter sequences of orthologous genes between these two diploid progenitors were not as conserved as the CDs sequences. For example, *MET-B* promoter contained a heat inducing CCAAT-box that was not detected in *MET-A* promoter. *MET-B* promoter contained a G-box but not in *MET-A* promoter. *ROS1-A* promoter didn't contain W-box that could be detected in *ROS1-B* promoter. Additionally, we found that most C5-MTase gene promoters contained cold inducing element but not in DNA demethylase gene promoters.

### Synteny analyses of C5-MTase and DNA demethylase genes in AA and BB genomes

A syntenic block is defined as the region in which three or more conserved homologs (BLASTP *E* < 10^−20^) were located within a 100 kb region between genomes (Sato et al., 2008). To further investigate the potential evolutionary mechanisms of C5-MTases and DNA demethylase genes, we performed all-vs.-all local BLASTP to identify synteny blocks in peanut genome. Blocks of 100 kb chromosome containing C5-MTases and DNA demethylase genes were used for synteny analysis.

**Figure 3:**
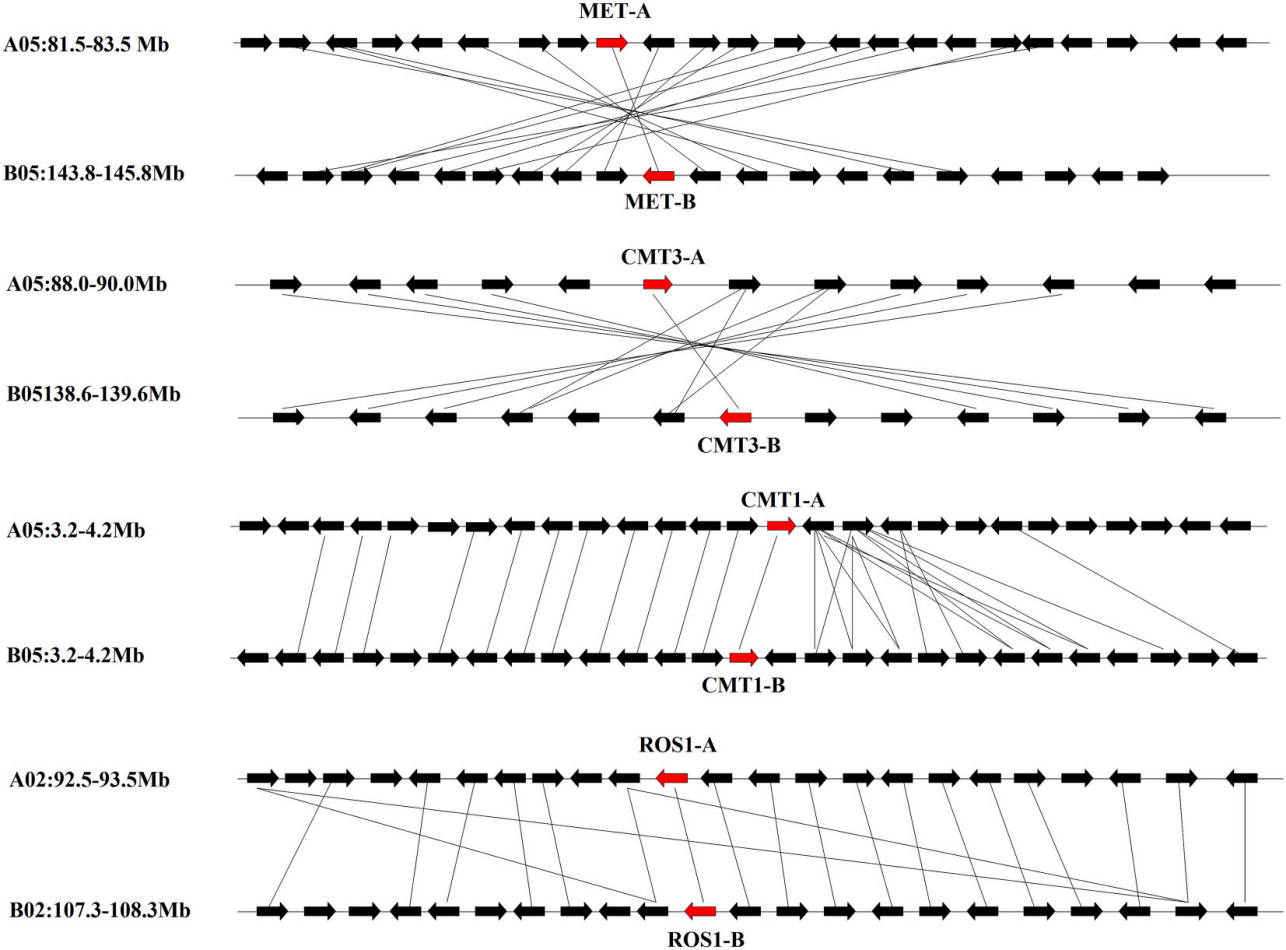
**Syntenic chromosomal segments between flanking genes from AA genome and their orthologous genes from BB genome**. Red arrows represent C5-MTase or demethylase gene, black arrows represent flanking genes.

Blocks from AA and from BB genomes were inter-genomic synteny blocks. Both in AA and BB wild peanut, no intra-genomic synteny blocks were found for C5-MTase or DNA demethylase genes. Twelve pairs of inter-genomic synteny blocks contained C5-MTase or DNA demethylase genes were found in peanut genome. Figure 3 showed that high level of synteny was maintained between AA and BB genomes.

### Selective pressure analysis of wild peanut C5-MTase and DNA demethylase genes

To detect whether different groups of wild peanut C5-MTases were under different selective pressure, the hypothesis testing on CMT and DRM2 groups of C5-MTase was performed using site models. Because MET group only contained two genes that belonged to one orthologous pair, they could not be analyzed using site models. M0 showed that wild peanut C5-MTase family underwent purifying selection (ω = 0.82896). The discrete model M3 fit better than one-ratio model M0, suggesting that ω ratios varied among sites (Table S8; LRT, *P* < 0.05). Although M1a vs. M2a comparison didn't find positive selection site with a *P* < 0.05, M7 vs. M8 comparison detected many positive selection sites with a 0.5 < *P* < 0.9. These positive selection sites in C5-MTase genes indicated functional diversity and structural variation between different members.

In wild peanut CMT group, M2a (positive selection model) identified only nine positive selection sites. However, M1a vs. M2a comparison showed that the result of M2a model was not reliable (*P* > 0.9). The M7 vs. M8 comparison identified 48 positive selection sites (*P* < 0.1). The M0 suggested that both CMT genes (ω = 0.65814) and DRM2 genes (ω = 0.44354) underwent purifying selection. Only one positively selected site was detected by M2a in wild peanut DRM2 group. These results suggested that wild peanut DRM2 genes were highly conserved and the majority of sites were under purifying selection (Table S8).

M0 result suggested that DNA demethylase genes underwent positive selection (ω = 1.12428). Both M1a vs. M2a comparison and M7 vs. M8 comparison detected more than 200 positive selection sites (*P* < 0.05). M3 didn't found positive selection site. However, M0 vs. M3 comparison showed that result of M3 model was not reliable (LRT, *P* > 0.9) (Table S8).

### 3D structural features and activity of BAH domain of peanut C5-MTases

**Figure 4:**
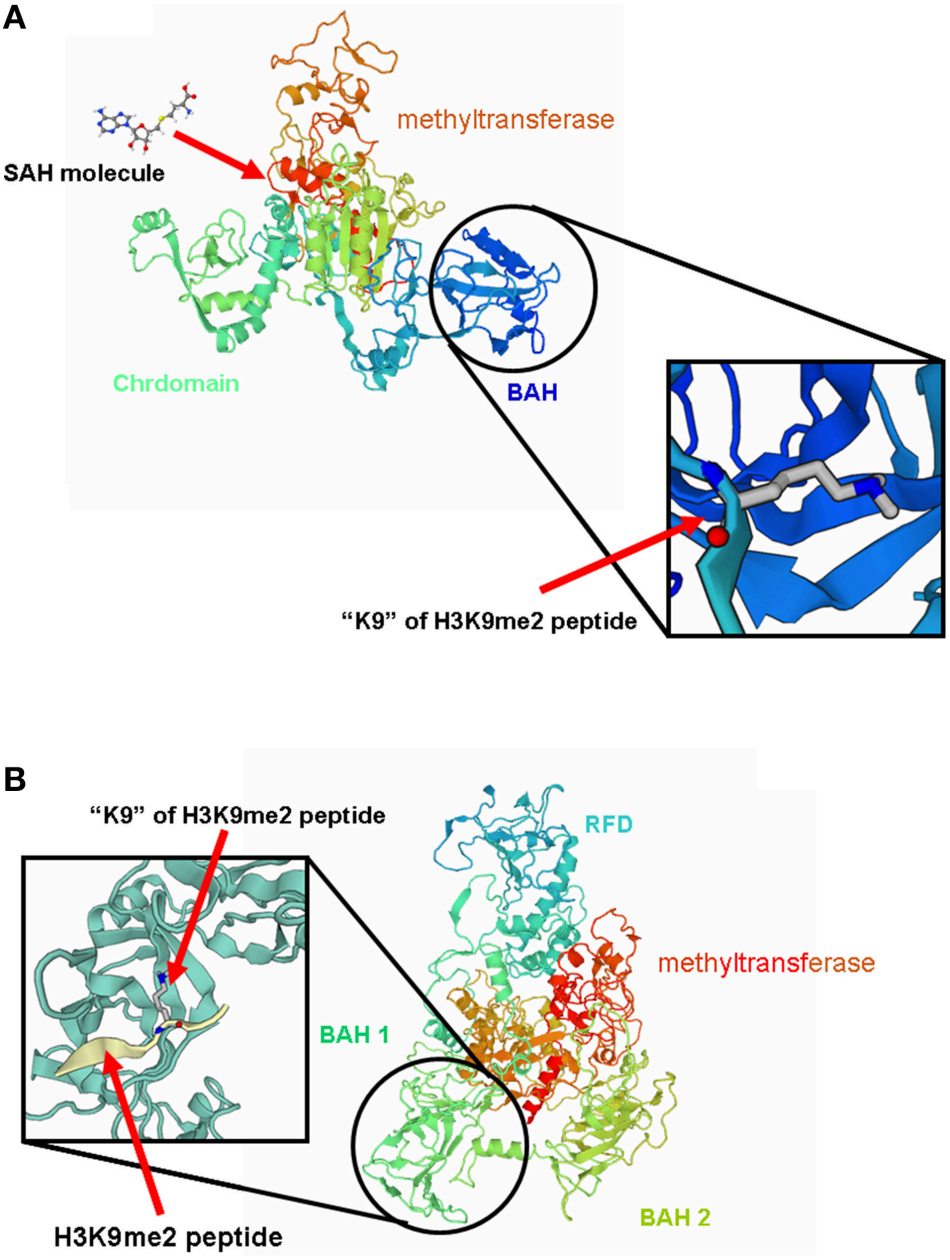
**3D structure of MET-B and CMT2-B**. **(A)** represents the 3D structure of MET-B and **(B)** represents the 3D structure of CMT2-B.

**Figure 5:**
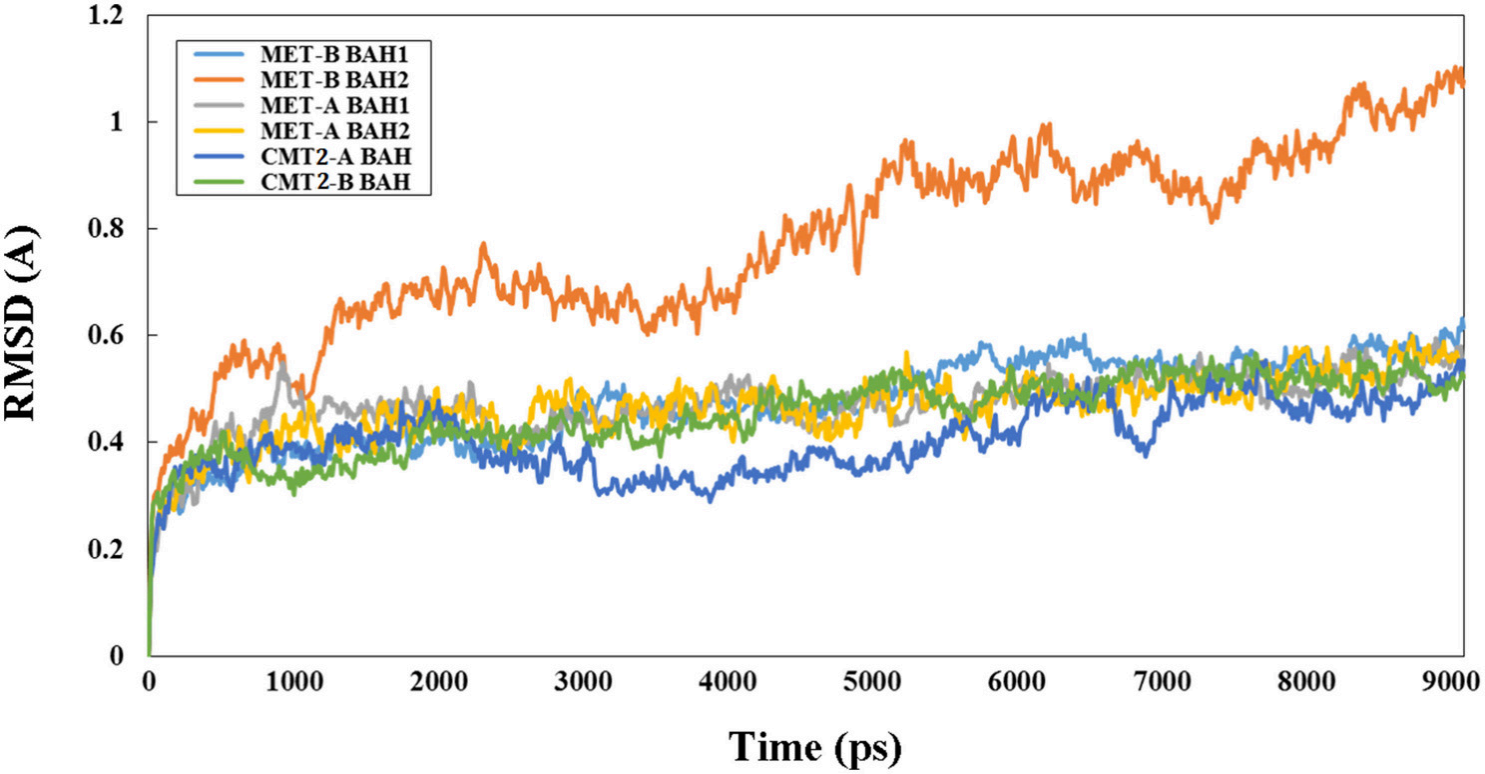
**RMSD of peanut B domain from MET and CMT group members**. “Y” axis represents RSDM (Ångstrom, A) and “X” axis represents time (picosecond, ps).

3D structure analysis of MET-A, MET-B, CMT2-A, and CMT2-B showed that peanut MET and CMT members having similar structure to members from other legume species (Garg et al., 2014). The methyltransferase domain (in red) of CMT2-B which could combine SAH and the BAH domain (in blue) of CMT2-B could combine H3K9 methylated-lysine (Figure 4). In Figure 4B, the methyltransferase domain (in red) of MET-B, the BAH1 domain (in green) of MET-B and the BAH2 domain (in yellow) were indicated. In other legume MET members, the BAH1 domains contained seven conserved amino acids which formed an aromatic cage, while BAH domain of CMT contained three conserved amino acids forming an aromatic cage. The aromatic cage could recognize and capture methylated-lysine (Garg et al., 2014). All BAH1 domains of peanut MET group members contained aromatic cages. For example, aromatic cage of MET-A contained seven amino acids: 775(D), 783(I), 784(Y), 785(F), 786(V), 788(Y), and 789(M). All BAH2 domains of peanut MET group members didn't contain aromatic cages. All BAH domains of peanut CMT group members contained 3 conserved amino acids. For example, these 3 amino acids are 449(Y), 454 (W), and 456 (Y) in CMT2-A. In order to investigate the difference between BAH2 and BAH1 or CMT-BAH, we analyzed the stability of these proteins within GROMACS filed. The RMSD of MET-B BAH2 was higher than MET-B BAH1 and other peanut CMT-BAH when they were in solution for 9 ns (Figure 5). Tracking the cavity gap motion by essential dynamic analysis showed collective motion of MET-A and MET-B BAH2 were aberrant comparing with MET-A BAH1, MET-B BAH1, and CMT-BAH. This result suggested that MET-B BAH2 was less stable than BAH1. Therefore, BAH1 could combine methylated-lysine better (Maia et al., 2012).

### Expression of C5-MTase and DNA demethylase genes in cultivated peanut

C5-MTase and DNA demethylase play important roles in plant development through regulating the level of DNA methylation (Ecker, 2013; Eichten et al., 2013; Candaele et al., 2014; Zhong et al., 2014). For example, the extensive de-methylation occurring in endosperm genome can active the expression of many genes (Gehring et al., 2009; Hsieh et al., 2009; Zemach et al., 2010; Wang et al., 2015).

From the genome sequences we identified MET, CMT1-3, DRM2, DRM2X1, DRM2X2, ROS1, ROS1-like, ROS1X2, DME, and DME-like gene in cultivated peanut. These gene sequences were similar to their orthologous genes from AA and BB genomes (data not shown). To gain insights into the putative function of these genes in cultivated peanut, the expression patterns of these genes were investigated by qRT-PCR analysis.

**Figure 6:**
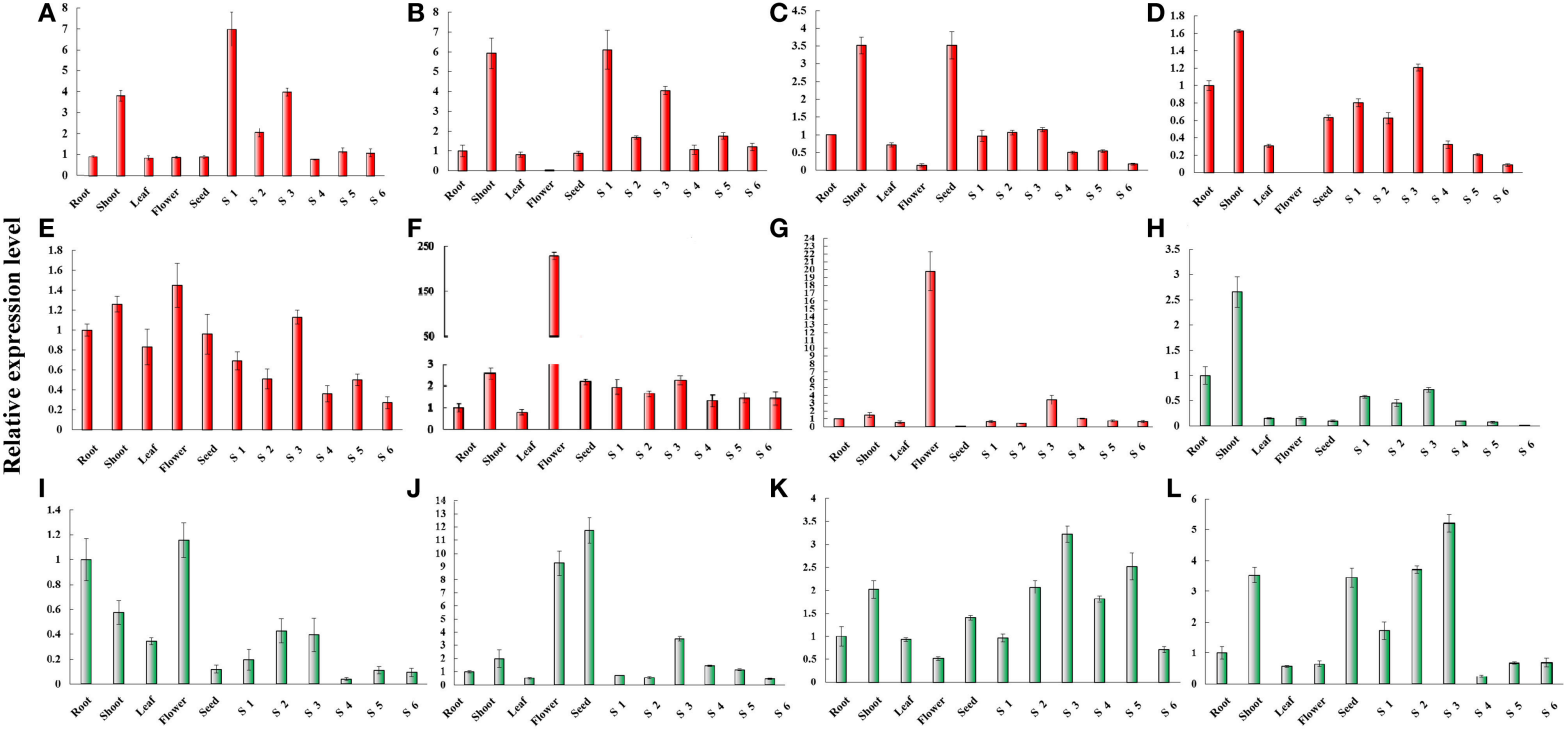
**Relative expression levels of peanut C5-MTase and demethylase genes in different tissue**. **(A–L)** Respectively, presents the relative expression levels of *MET, CMT1, CMT2, CMT3, DRM2, DRM2X1, DRM2X2, DME, DME-like, ROS1, ROS2-like*, and *ROS1X2* in different tissues. “Y” axis represents relative expression level and “X” axis represents tissue names.

The expression of *MET* was predominant detected in shoot and gynophore. The expression level of *MET* was the highest in Stage 1 gynophores (S1). The expression of *CMT1* was also predominantly detected in shoot and gynophore. The expression of *CMT2* was high in shoot and seed. *CMT3* was expressed highly in shoot, root, and gynophore. The expression level of *CMT3* was declined from S3 to S6 in the gynophore apex. The expression of *DRM2* was ubiquitously in all tissues analyzed. Both *DRM2X1* and *DRM2X2* were strongly expressed in flowers (Figure 6). *DME* was predominantly expressed in shoot and root. The expression level of *DME*-like was high in root, shoot, leaf, flower, S2, and S3. *ROS1* was highly expressed in flower and seed. *ROS1-like* and *ROS1X2* showed a similar expression patterns in different tissues (Figure 6). Interestingly, the expression level of *ROS1, ROS1-like*, and *ROS1X2* increased from S1 to S3. From S1 to S3, gynophores experienced the transition from light to dark condition and the quiescent pre-embryo started to develop (Xia et al., 2013). The expression levels of these genes during S1 to S3 were consistent with RNA-seq results. In addition, RNA-seq result showed that *DME-like* and *DRM2X2* were expressed at a very low level during S1 and S2 (Figure S6). Abiotic stress like light, high, and low temperature, PEG treatment and cold all could affect DNA methylation dynamic in plant genome. In this study, we investigated the expression patterns of cultivated peanut C5-MTases and DNA demethylase genes under PEG treatment, high, and low temperature conditions by qRT-PCR (Figures S7–S9).

### Subcellular localization of wild and cultivated peanut C5-MTases and DNA demethylases

**Figure 7:**
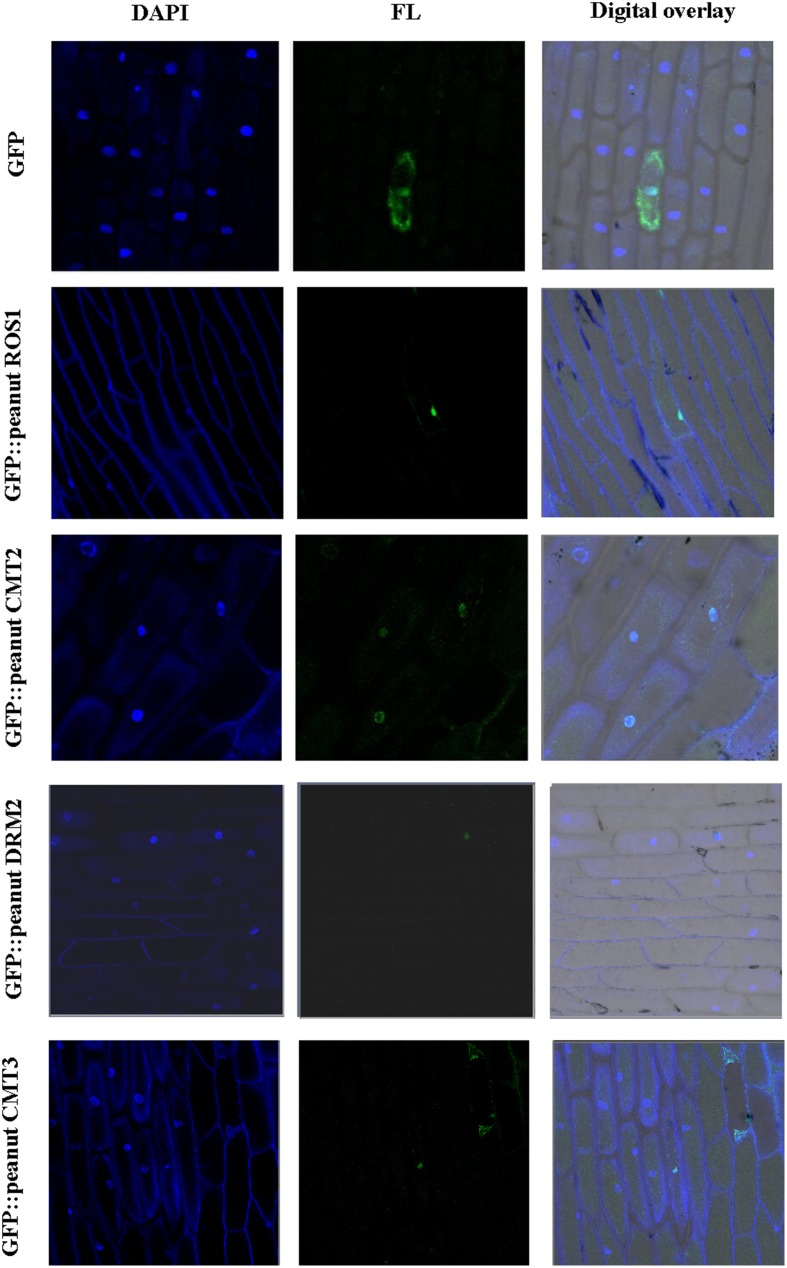
**Subcellular localization of peanut C5-MTases and demethylases**. Visualization of GFP fused peanut DRM2, CMT2, CMT3, and ROS1 as compared to pBI221-GFP control vector in onion epidermal cells. FL represents GFP fluorescence; DAPI represents DAPI staining.

The presence of nuclear localization signal (NLS) was predicted in wild peanut C5-MTases and DNA demethylases (Table S9). Monopartite NLS was predicted in DRM2X2-A, DRM2X2-B, CMT2-A, CMT2-B, DME-like-A, DME-like-B, ROS1X2-A, ROS1X2-B, and ROS1-like-A and bipartite NLS was predicted in other proteins. The amino acid sequences of the NLSs between orthologous were conserved except ROS1-like-A and ROS1-like-B. NLSs of DRM2X1-A, DRM2X1-B, DRM2X2-A, DRM2X2-B, CMT3-A, and DME-like-B were relatively weak (score < 5). In *C. arietinum, O. sativa*, and *S. lycopersicum*, CMT and DRM proteins have been confirmed localized in nucleus (Wada et al., 2003; Dangwal et al., 2013; Cao et al., 2014; Garg et al., 2014). We studied the subcellular localization of peanut C5-MTases and DNA demethylases. Cultivated peanut CMT2, CMT3, DRM2, and ROS1 were cloned in pBI221 vector with C-terminal GFP fusion. These fusion proteins were transiently expressed in onion epidermal cells. The results showed that these GFP fusion proteins were all localized in nucleus (Figure 7).

## Discussion

In wild peanut genomes, 14 C5-MTases and 10 DNA demethylases were found, including 12 pairs of orthologous genes. The sequences of these C5-MTase and DNA demethylase proteins or genes were similar to their orthologous. These genes and their orthologous were located in the same chromosome. Synteny analyses showed that the segments contained C5-MTase or DNA demethylase genes were very similar with the segments contained their orthologous genes. Promoter analysis showed that the promoters of most C5-MTase and DNA demethylase genes contained light-, heat-, cold-, and salt-induce cis-regulated elements.

Both AA and BB wild peanut genome contained three groups of C5-MTase genes: MET, CMT, and DRM2 group. Unlike *Arabidopsis*, peanut AA genome and BB genome only contained one MET1 gene, *MET-A* and *MET-B* (Hsieh et al., 2011). Like other plant, peanut CMT group contained CMT1, CMT2, and CMT3 type genes (Garg et al., 2014). CMT1-A, CMT2-A, and CMT3-A genes were located in AA genome, while CMT1-B, CMT2-B, and CMT3-B genes were in BB genome. Previous study showed that DRM2 requires intact UBA domain during RdDM in *Arabidopsis* (Henderson et al., 2010). However, some wild peanut DRM2 group members didn't contained UBA domain such as DRM2-A, DRM2-B, DRM2X1-A, and DRM2X1-B. In seed plant and mammalian, formation of DRM2 homodimer or heterodimer was required to direct DNA *de novo* methylation (Zhong et al., 2014). It is possible that the DRM2 group member without UBA domain could direct DNA *de novo* methylation upon forming heterodimer with the DRM2 group member with UBA domain. N-terminus of MET harbor two BAH domains, which might act as a site for protein-protein interaction (Garg et al., 2014). We predicted the 3D structures of wild peanut C5-MTases. We found the structure variation of different BAH domains. We identified that BAH1 of peanut MET group members are similar to BAH domain of CMTs. Both BAH1 and CMT-BAH domains contained aromatic cages which might be involved in interaction of MET with methylated histones tails. BAH2 domains didn't contain aromatic cages. GROMOCS software analysis showed that the RMSD of BAH2 was higher than BAH1 and other CMT-BAH, and collective motion of BAH2 were aberrant comparing with BAH1 and CMT-BAH. The results suggested that BAH2 was less stable than BAH1.

The selective pressure analysis showed that wild peanut C5-MTase genes mainly underwent purifying selection in evolution but many positive selection sites could be detected. DNA demethylase genes mainly underwent positive selection. These results suggested that evolution of these genes was tightly associated with the environment change during peanut evolution history.

Genes encoding enzymes for DNA methylation maintenance, for example *CMT, CMT2*, and *CMT3* didn't express in flower. While genes encoding enzymes for *de novo* DNA methylation, for example, *DRM2X1* (not containing UBA domain) and *DRM2X2* (containing UBA domain) expressed primarily in flower. Previous studies have been proven that DRM containing UBA domain exhibited *de novo* methylation activity. Additionally, DNA methylation was reprogrammed in germ tissues or cells (Seisenberger et al., 2012). So, the silence of DNA methylation maintenance genes and activation of *de novo* methylation genes may be associated with DNA methylation reprogramming in peanut flower.

Peanut flowering and pollinated above ground. The fertilized ovary elongated and form a gynophore which also was called “peg” like structure which growth downward to the soil to complete pod development. We designate the above ground downward growing gynophore as stage 1 (S1). Gynophore that buried into soil for about 3 d is designated as stage 2 (S2) which is white in color and the pod enlargement is not observed. Peg that buried in soil for 9 d is white in color with enlarged pod is designated as stage 3 (S3). The expression level of DNA demethylase genes (*ROS1, ROS1-like*, and *ROS1X2)* increased from S1 to S3. From S1 to S3, the growth condition of gynophores changed from light to dark condition. Recently, epigenetic modifications have been shown to play crucial roles in response to environmental stimuli (Chinnusamy and Zhu, 2009; Gutzat and Mittelsten Scheid, 2012). The drastic change of the environment could be the cause of changed expression of these genes. The changed expression of these genes could affect the methylation pattern of peanut gynophore. Our results of methylation analysis showed that the methylation patterns were significantly different among S1, S2, and S3 (data not shown). In maize embryo and endosperm, the expression change of methylation enzymes affected the methylation patterns of these two tissues and led to distinct developmental fates (Wang et al., 2015). There is the possibility that the development of peanut pod and embryo could be regulated by methylation. DEG data showed that another DNA demethylase gene *DME-like* didn't express in S1 and S2. In *Arabidopsis*, different DNA demethylases directed DNA de-methylation in different tissues or cells, for example, AtDME directed DNA de-methylation only in central cell and synergids (Zhu, 2009).

Heat, PEG treatment and cold stress could affect DNA methylation dynamic in plants (Gong et al., 2002; Naydenov et al., 2015). We analyzed the transcript abundance of MTase genes under abiotic stress conditions in cultivated peanut. The results showed that cold, heat or PEG stress could influence the expression level of cultivated peanut C5-MTase and DNA demethylase genes. Higher transcript levels of genes during abiotic stress suggested their involvement in stress induced DNA methylation changes. For example, the expressions of most cultivated peanut C5-MTase genes were increased after 3 or 6 h of cold treatment. DME-like gene showed an increased expression after 48 h of cold treatment. The expression of most C5-MTase genes increased after PEG treatment. These results suggested that these genes might be involved in abiotic stress responses in cultivated peanut.

We studied the subcellular localization of wild and cultivated peanut C5-MTases and DNA demethylase. The results showed that peanut C5-MTases were located in nucleus which was consisted with other species (Cao et al., 2014; Garg et al., 2014). We also found that cultivated peanut DNA demethylase ROS1 located in nucleus.

## Conclusions

We have identified members of C5-MTases and DNA demethylases in wild and cultivated peanut. We found differential transcript abundance of C5-MTase and DNA demethylase genes in different tissues and different developmental stages. Our study provided evidence for the roles of C5-MTases and DNA demethylases in response to environmental stresses in cultivated peanut. These results are useful for better understanding the complexity of these two gene families, and will facilitate epigenetic studies in peanut.

## Author contributions

XW and SW designed the study, wrote the manuscript and finalized the figures and tables. PW and CG carried out most of the experiment, data analysis, and wrote the method section of the manuscript. XB, SZ, CZ, HX, HS, LH, performed experiments and took care of the plants.

### Conflict of interest statement

The authors declare that the research was conducted in the absence of any commercial or financial relationships that could be construed as a potential conflict of interest.

## Supplementary material

Figure S1 **HhH-GPD domain in demethylase**.

Figure S2 **Motifs identified in C5-MTases and demethylases. (A)** Motifs of C5-MTases and **(B)** Motifs of demethylases.

Figure S3 **Six conserved motifs in C5-MTases. (A)** Six conserved motifs of MET group, **(B)** Six conserved motifs of CMT group, and **(C)** Six conserved motifs of DRM2 groups.

Figure S4 **Neighbor-joining phylogeny of demethylases**.

Figure S5 **Gene structure of peanut C5-MTases and demethylases. (A)** Gene structure of C5-MTases and **(B)** Gene structure of demethylases.

Figure S6 **Heat map (using RPKM value) of C5-MTase and demethylase genes in different cultivated peanut tissue**.

Figure S7 **Relative expression levels of cultivated peanut C5-MTase and demethylase genes under cold stress. (A–L)** respectively represent the relative expression levels of *MET, CMT1, CMT2, CMT3, DRM2, DRM2X1, DRM2X2, DME, DME-LIKE, ROS1, ROS2-like*, and *ROS1X2* under cold stress from 0 to 48 h.“Y” axis represents relative expression level and “X” axis represents treated time (hour, h).

Figure S8 **Relative expression levels of cultivated peanut C5-MTase and demethylase genes under PEG stress. (A–L)** respectively represent the relative expression levels of *MET, CMT1, CMT2, CMT3, DRM2, DRM2X1, DRM2X2, DME, DME-like, ROS1, ROS2-like*, and *ROS1X2* under PEG treatment from 0 to 48 h. “Y” axis represents relative expression level and “X” axis represents treated time (hour, h).

Figure S9 **Relative expression levels of cultivated peanut C5-MTase and demethylase genes under heat stress. (A–L)** respectively represent the relative expression levels of *MET, CMT1, CMT2, CMT3, DRM2, DRM2X1, DRM2X2, DME, DME-like, ROS1, ROS2-like*, and *ROS1X2* under heat treatment from 0 to 48 h. “Y” axis represents relative expression level and “X” axis represents treated time (hour, h).

Table S1 **C5-MTase and demethylase genes identified in other species**.

Table S2 **Conserved motif sequences of C5-MTases and demethylases**.

Table S3 **Primers of cultivated peanut C5-MTase and demethylase genes for qRT-PCR**.

Table S4 **Primers of cultivated peanut C5-MTase and demethylase genes for gene cloning**.

Table S5 **Domains and their positions in wild peanut C5-MTases and demethylase proteins**.

Table S6 **Identity of wild peanut C5-MTase and DNA demethylase orthologous pairs**.

Table S7 **Cis-acting regulatory elements of wild peanut C5-MTase and demethylase gene promoters**.

Table S8 **Likelihood values and parameter estimates for wild peanut C5-MTase and DNA demethylase family genes**.

Table S9 **Nuclear localization signal in wild peanut C5-MTase and DNA demethylase genes**.

## References

[B1] Bailey T. L. Williams N. Misleh C. Li W. W. (2006). MEME: discovering and analyzing DNA and protein sequence motifs. Nucleic. Acids. Res. 34(Suppl. 2), W369–W373. 10.1093/nar/gkl198 16845028 PMC1538909

[B2] Bender J. (2004). DNA methylation and epigenetics. Annu. Rev. Plant. Biol. 55, 41–68. 10.1146/annurev.arplant.55.031903.141641 15725056

[B3] Candaele J. Demuynck K. Mosoti D. Beemster G. T. Inzé D. Nelissen H. (2014). Differential methylation during maize leaf growth targets developmentally regulated genes. Plant Physiol. 164, 1350–1364. 10.1104/pp.113.233312 24488968 PMC3938625

[B4] Cao D. Ju Z. Gao C. Mei X. Fu D. Zhu H. (2014). Genome-wide identification of cytosine-5 DNA methyltransferases and demethylases in *Solanum lycopersicum*. Gene 550, 230–237. 10.1016/j.gene.2014.08.034 25149677

[B5] Chen F. Li Q. Sun L. He Z. (2006). The rice 14-3-3 gene family and its involvement in responses to biotic and abiotic stress. DNA. Res. 13, 53–63. 10.1093/dnares/dsl001 16766513

[B6] Chinnusamy V. Zhu J. K. (2009). Epigenetic regulation of stress responses in plants. Curr. Opin. Plant. Biol. 12, 133–139. 10.1016/j.pbi.2008.12.006 19179104 PMC3139470

[B7] Dangwal M. Malik G. Kapoor S. Kapoor M. (2013). *De novo* methyltransferase, OsDRM2, interacts with the ATP-dependent RNA helicase, OseIF4A, in rice. J. Mol. Biol. 425, 2853–2866. 10.1016/j.jmb.2013.05.021 23732981

[B8] Du J. Zhong X. Bernatavichute Y. V. Stroud H. Feng S. Caro E.. (2012). Dual binding of chromomethylase domains to H3K9me2-containing nucleosomes directs DNA methylation in plants. Cell 151, 167–180. 10.1016/j.cell.2012.07.034 23021223 PMC3471781

[B9] Ecker J. R. (2013). Epigenetic trigger for tomato ripening. Nat. Biotechnol. 31, 119–120. 10.1038/nbt.2497 23392509 PMC5215857

[B10] Eichten S. R. Briskine R. Song J. Li Q. Swanson-Wagner R. Hermanson P. J.. (2013). Epigenetic and genetic influences on DNA methylation variation in maize populations. Plant Cell 25, 2783–2797. 10.1105/tpc.113.114793 23922207 PMC3784580

[B11] Feinberg A. P. (2007). Phenotypic plasticity and the epigenetics of human disease. Nature 447, 433–440. 10.1038/nature05919 17522677

[B12] Freitas F. O. Moretzsohn M. C. Valls J. F. (2007). Genetic variability of Brazilian Indian landraces of *Arachis hypogaea* L. Genet. Mol. Res. 6, 675–684. 18050088

[B13] Garg R. Kumari R. Tiwari S. Goyal S. (2014). Genomic survey, gene expression analysis and structural modeling suggest diverse roles of DNA methyltransferases in legumes. PLoS ONE. 9:e88947. 10.1371/journal.pone.0088947 24586452 PMC3934875

[B14] Gehring M. Bubb K. L. Henikoff S. (2009). Extensive demethylation of repetitive elements during seed development underlies gene imprinting. Science 324, 1447–1451. 10.1126/science.1171609 19520961 PMC2886585

[B15] Gong Z. Morales-Ruiz T. Ariza R. R. Roldán-Arjona T. David L. Zhu J. K. (2002). ROS1, a repressor of transcriptional gene silencing in Arabidopsis, encodes a DNA glycosylase/lyase. Cell 111, 803–814. 10.1016/s0092-8674(02)01133-9 12526807

[B16] Guo A.-Y. Zhu Q.-H. Chen X. Luo J.-C. (2007). GSDS: a gene structure display server. Yi Chuan 29, 1023–1026. 17681935

[B17] Gutzat R. Mittelsten Scheid O. (2012). Epigenetic responses to stress: triple defense. Curr. Opin. Plant. Biol. 15, 568–573. 10.1016/j.pbi.2012.08.007 22960026 PMC3508409

[B18] Heard E. Disteche C. M. (2006). Dosage compensation in mammals: fine-tuning the expression of the X chromosome. Genes. Dev. 20, 1848–1867. 10.1101/gad.1422906 16847345

[B19] Henderson I. R. Deleris A. Wong W. Zhong X. Chin H. G. Horwitz G. A.. (2010). The *de novo* cytosine methyltransferase DRM2 requires intact UBA domains and a catalytically mutated paralog DRM3 during RNA-directed DNA methylation in *Arabidopsis thaliana*. PLoS.Genet. 6:e1001182. 10.1371/journal.pgen.1001182 21060858 PMC2965745

[B20] Hsieh T. F. Ibarra C. A. Silva P. Zemach A. Eshed-Williams L. Fischer R. L.. (2009). Genome-wide demethylation of Arabidopsis endosperm. Science 324, 1451–1454. 10.1126/science.1172417 19520962 PMC4044190

[B21] Hsieh T. F. Shin J. Uzawa R. Silva P. Cohen S. Bauer M. J.. (2011). Regulation of imprinted gene expression in Arabidopsis endosperm. Proc. Natl. Acad. Sci. U.S.A. 108, 1755–1762. 10.1073/pnas.1019273108 21257907 PMC3033266

[B22] Kim M. Ohr H. Lee J. W. Hyun Y. Fischer R. L. Choi Y. (2008). Temporal and spatial downregulation of Arabidopsis MET1 activity results in global DNA hypomethylation and developmental defects. Mol. Cells. 26, 611–615. 18820427 PMC4109710

[B23] Klose R. J. Bird A. P. (2006). Genomic DNA methylation: the mark and its mediators. Trends. Biochem. Sci. 31, 89–97. 10.1016/j.tibs.2005.12.008 16403636

[B24] Kochert G. Stalker H. T. Gimenes M. Galgaro L. Romero-Lopes C. Moore K. (1996). RFLP and cytogenetic evidence on the origin and evolution of allotetraploid domesticated peanut, *Arachis hypogaea* (Leguminosae). Am. J. Bot. 83, 1282–1291

[B25] Krzywinski M. Schein J. Birol I. Connors J. Gascoyne R. Horsman D.. (2009). Circos: an information aesthetic for comparative genomics. Genome. Res. 19, 1639–1645. 10.1101/gr.092759.109 19541911 PMC2752132

[B26] Law J. A. Jacobsen S. E. (2010). Establishing, maintaining and modifying DNA methylation patterns in plants and animals. Nat. Rev. Genet. 11, 204–220. 10.1038/nrg2719 20142834 PMC3034103

[B27] Lin Y. Cheng Y. Jin J. Jin X. Jiang H. Yan H.. (2014). Genome duplication and gene loss affect the evolution of heat shock transcription factor genes in legumes. PLoS ONE. 9:e102825. 10.1371/journal.pone.0102825 25047803 PMC4105503

[B28] Lu S. C. Mato J. M. (2012). S-adenosylmethionine in liver health, injury, and cancer. Physiol. Rev. 92, 1515–1542. 10.1152/physrev.00047.2011 23073625 PMC3698976

[B29] Maia A. M. da Silva J. H Mencalha A. L. Caffarena E. R. Abdelhay E. (2012). Computational modeling of the bHLH domain of the transcription factor TWIST1 and R118C, S144R and K145E mutants. BMC Bioinformatics 13:184. 10.1186/1471-2105-13-184 22839202 PMC3507644

[B30] Malik G. Dangwal M. Kapoor S. Kapoor M. (2012). Role of DNA methylation in growth and differentiation in *Physcomitrella patens* and characterization of cytosine DNA methyltransferases. FEBS. J. 279, 4081–4094. 10.1111/febs.12002 22943564

[B31] Malone T. Blumenthal R. M. Cheng X. (1995). Structure-guided analysis reveals nine sequence motifs conserved among DNA aminomethyltransferases, and suggests a catalytic mechanism for these enzymes. J. Mol. Biol. 253, 618–632. 7473738 10.1006/jmbi.1995.0577

[B32] Matzke M. A. Mosher R. A. (2014). RNA-directed DNA methylation: an epigenetic pathway of increasing complexity. Nat. Rev. Genet. 15, 394–408. 10.1038/nrg3683 24805120

[B33] Moretzsohn M. C. Gouvea E. G. Inglis P. W. Leal-Bertioli S. C. M. Valls J. F. M. Bertioli D. J. (2013). A study of the relationships of cultivated peanut (*Arachis hypogaea*) and its most closely related wild species using intron sequences and microsatellite markers. Ann. Bot. 111, 113–126. 10.1093/aob/mcs237 23131301 PMC3523650

[B34] Naydenov M. Baev V. Apostolova E. Gospodinova N. Sablok G. Gozmanova M.. (2015). High-temperature effect on genes engaged in DNA methylation and affected by DNA methylation in Arabidopsis. Plant. Physiol. Biochem. 87, 102–108. 10.1016/j.plaphy.2014.12.022 25576840

[B35] Noy-Malka C. Yaari R. Itzhaki R. Mosquna A. Auerbach Gershovitz N. Katz A.. (2014). A single CMT methyltransferase homolog is involved in CHG DNA methylation and development of *Physcomitrella patens*. Plant. Mol. Biol. 84, 719–735. 10.1007/s11103-013-0165-6 24370935

[B36] Penterman J. Zilberman D. Huh J. H. Ballinger T. Henikoff S. Fischer R. L. (2007). DNA demethylation in the Arabidopsis genome. Proc. Natl. Acad. Sci. U.S.A. 104, 6752–6757. 10.1073/pnas.0701861104 17409185 PMC1847597

[B37] Rai K. Huggins I. J. James S. R. Karpf A. R. Jones D. A. Cairns B. R. (2008). DNA demethylation in zebrafish involves the coupling of a deaminase, a glycosylase, and gadd45. Cell 135, 1201–1212. 10.1016/j.cell.2008.11.042 19109892 PMC2629358

[B38] Sato S. Nakamura Y. Kaneko T. Asamizu E. Kato T. Nakao M.. (2008). Genome structure of the legume, *Lotus japonicus*. DNA. Res. 15, 227–239. 10.1093/dnares/dsn008 18511435 PMC2575887

[B39] Seisenberger S. Andrews S. Krueger F. Arand J. Walter J. Santos F.. (2012). The dynamics of genome-wide DNA methylation reprogramming in mouse primordial germ cells. Mol. Cell. 48, 849–862. 10.1016/j.molcel.2012.11.001 23219530 PMC3533687

[B40] Sharma R. Priya P. Jain M. (2013). Modified expression of an auxin-responsive rice CC-type glutaredoxin gene affects multiple abiotic stress responses. Planta 238, 871–884. 10.1007/s00425-013-1940-y 23918184

[B41] Slotkin R. K. Vaughn M. Borges F. Tanurdžić M. Becker J. D. Feijó J. A.. (2009). Epigenetic reprogramming and small RNA silencing of transposable elements in pollen. Cell 136, 461–472. 10.1016/j.cell.2008.12.038 19203581 PMC2661848

[B42] Tamura K. Dudley J. Nei M. Kumar S. (2007). MEGA4: Molecular Evolutionary Genetics Analysis (MEGA) software version 4.0. Mol. Biol. Evol. 24, 1596–1599. 10.1093/molbev/msm092 17488738

[B43] Van Der Spoel D. Lindahl E. Hess B. Groenhof G. Mark A. E. Berendsen H. J. (2005). GROMACS: fast, flexible, and free. J. Comput. Chem. 26, 1701–1718. 10.1002/jcc.20291 16211538

[B44] Wada Y. Ohya H. Yamaguchi Y. Koizumi N. Sano H. (2003). Preferential *de novo* methylation of cytosine residues in non-CpG sequences by a domains rearranged DNA methyltransferase from tobacco plants. J. Biol. Chem. 278:42386–42393. 10.1074/jbc.M303892200 12917429

[B45] Wang P. Xia H. Zhang Y. Zhao S. Zhao C. Hou L.. (2015). Genome-wide high-resolution mapping of DNA methylation identifies epigenetic variation across embryo and endosperm in Maize (*Zea may*). BMC Genomics 16:21. 10.1186/s12864-014-1204-7 25612809 PMC4316406

[B46] Wu L. Zhou H. Zhang Q. Zhang J. Ni F. Liu C.. (2010). DNA methylation mediated by a microRNA pathway. Mol. Cell. 38, 465–475. 10.1016/j.molcel.2010.03.008 20381393

[B47] Xia H. Zhao C. Hou L. Li A. Zhao S. Bi Y.. (2013). Transcriptome profiling of peanut gynophores revealed global reprogramming of gene expression during early pod development in darkness. BMC Genomics 14:517 10.1186/1471-2164-14-517 23895441 PMC3765196

[B48] Xiao W. Custard K. D. Brown R. C. Lemmon B. E. Harada J. J. Goldberg R. B.. (2006). DNA methylation is critical for Arabidopsis embryogenesis and seed viability. Plant Cell 18, 805–814. 10.1105/tpc.105.038836 16531498 PMC1425851

[B49] Yang Z. (2007). PAML 4: phylogenetic analysis by maximum likelihood. Mol. Biol. Evol. 24, 1586–1591. 10.1093/molbev/msm088 17483113

[B50] Yang Z. Nielsen R. Goldman N. Pedersen A. M. (2000). Codon-substitution models for heterogeneous selection pressure at amino acid sites. Genetics 155, 431–449. 10790415 10.1093/genetics/155.1.431 PMC1461088

[B51] Yang Z. Wong W. S. Nielsen R. (2005). Bayes empirical bayes inference of amino acid sites under positive selection. Mol. Biol. Evol. 22, 1107–1118. 10.1093/molbev/msi097 15689528

[B52] Zemach A. Kim M. Y. Silva P. Rodrigues J. A. Dotson B. Brooks M. D.. (2010). Local DNA hypomethylation activates genes in rice endosperm. Proc. Natl. Acad. Sci. U.S.A. 107, 18729–18734. 10.1073/pnas.1009695107 20937895 PMC2972920

[B53] Zhong X. Du J. Hale C. J. Gallego-Bartolome J. Feng S. Vashisht A. A.. (2014). Molecular mechanism of action of plant DRM *de novo* DNA methyltransferases. Cell 157, 1050–1060. 10.1016/j.cell.2014.03.056 24855943 PMC4123750

[B54] Zhu J. K. (2009). Active DNA demethylation mediated by DNA glycosylases. Annu. Rev. Genet. 43, 143–166. 10.1146/annurev-genet-102108-134205. 19659441 PMC3137514

