# Supplementary figures and images for: Genome-Wide Identification and Comparative Analysis of Cytosine-5 DNA Methyltransferase and Demethylase Families in Wild and Cultivated Peanut
Source: Front Plant Sci. 2016 Feb 3;7:7. doi: 10.3389/fpls.2016.00007 (PMC4737905; doi:10.3389/fpls.2016.00007)

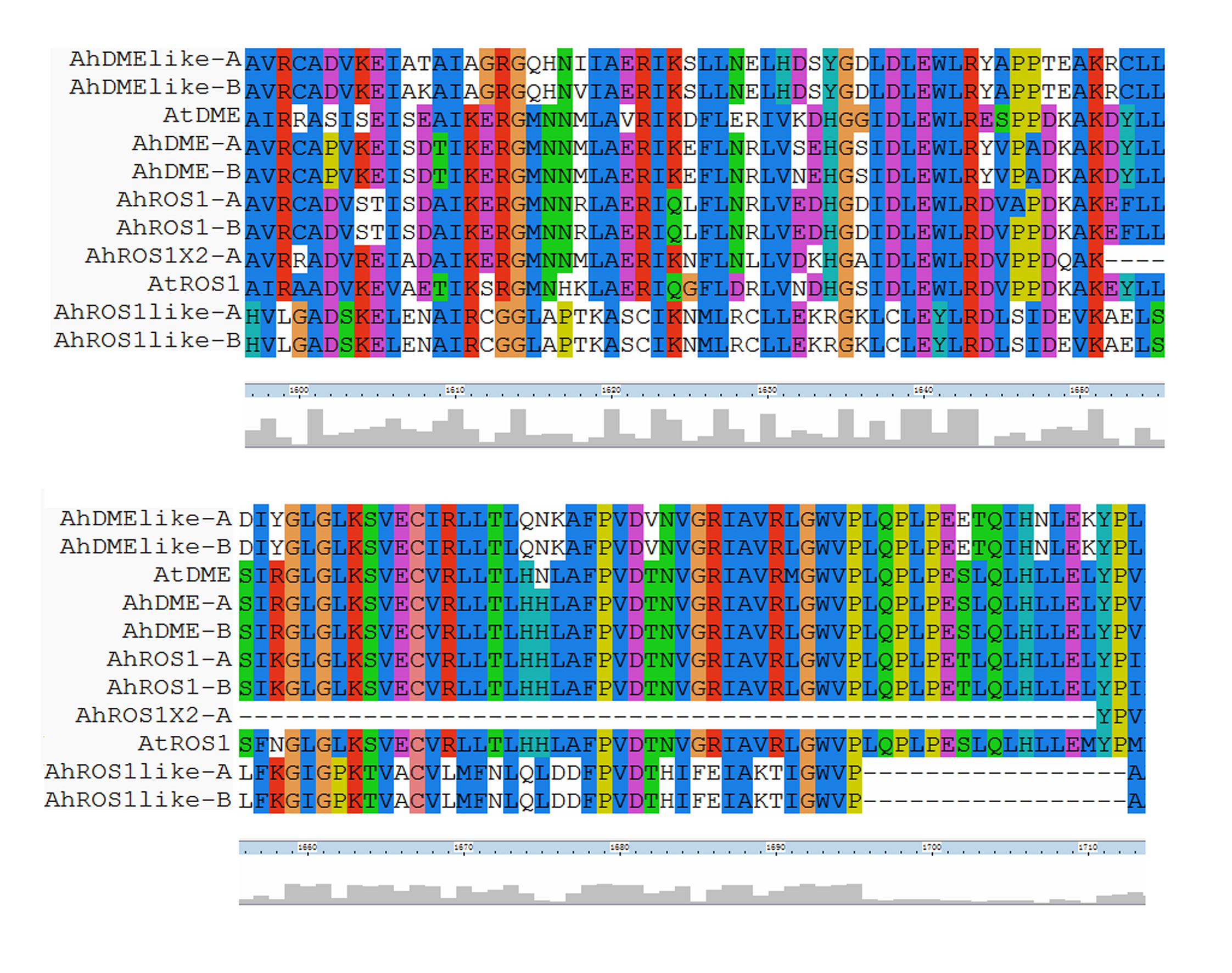

Supplement: Figure S1 — HhH-GPD domain in demethylase. [file Image1.TIF]

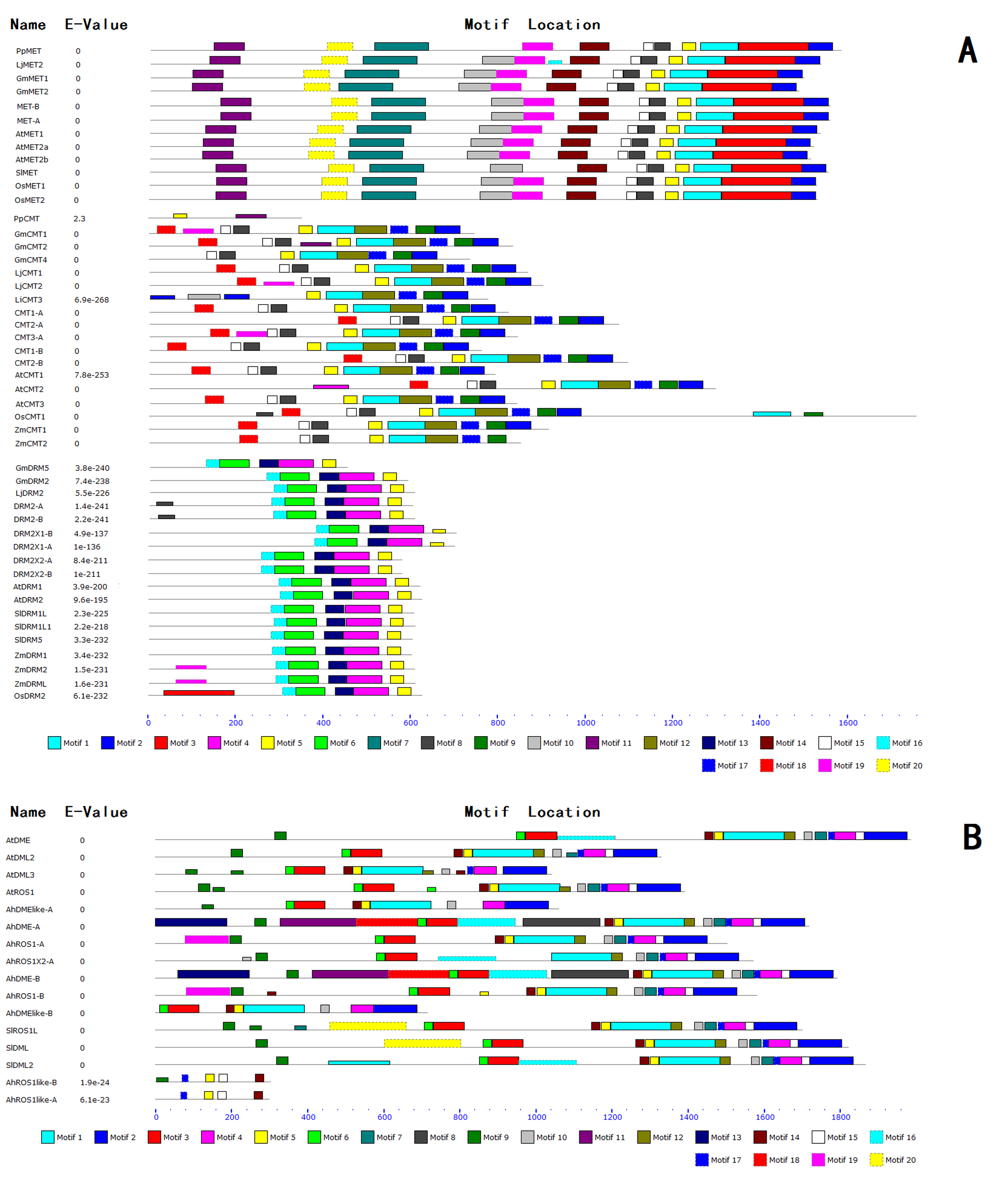

Supplement: Figure S2 — Motifs identified in C5-MTases and demethylases. (A) Motifs of C5-MTases and (B) Motifs of demethylases. [file Image2.TIF]

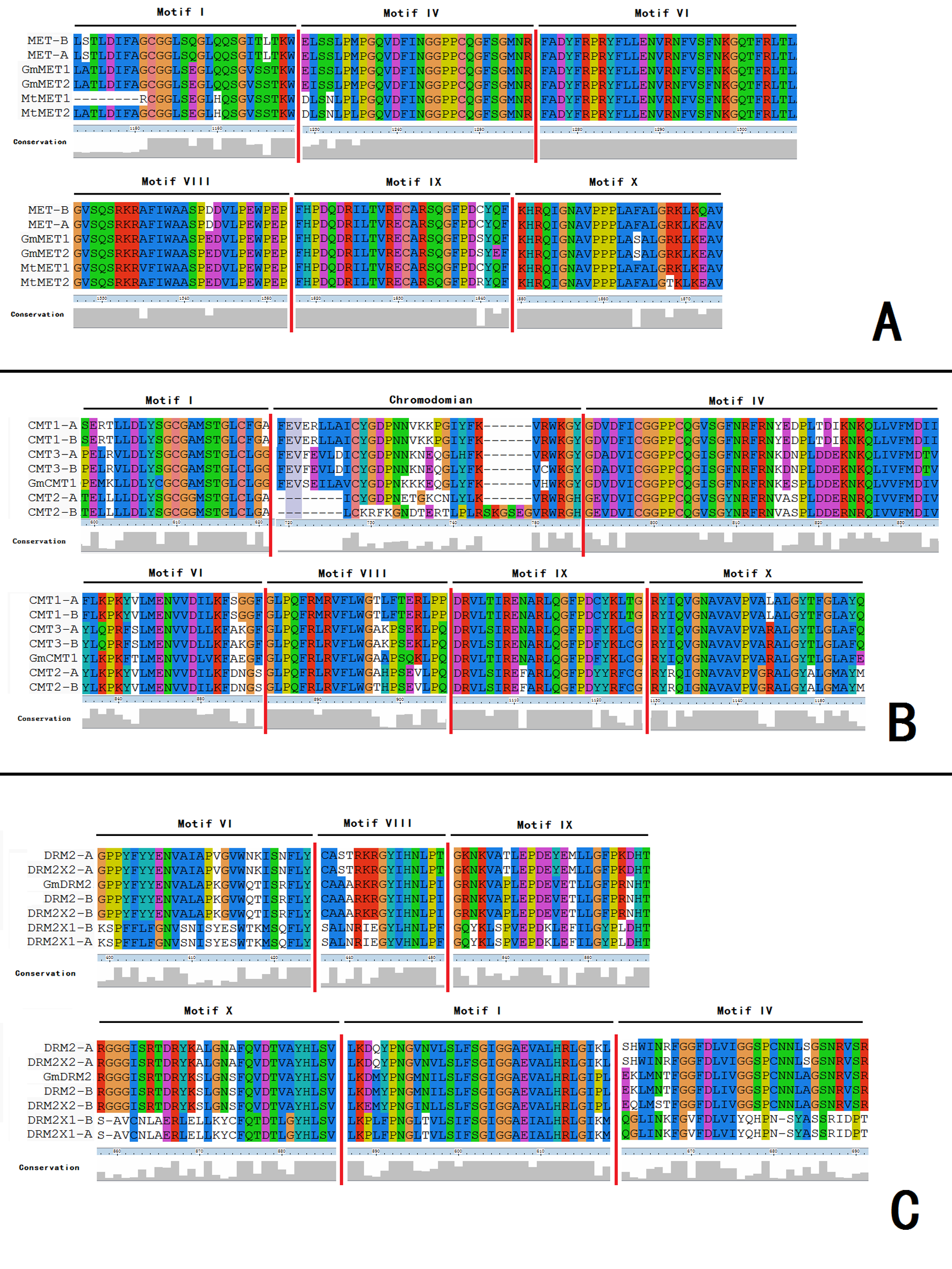

Supplement: Figure S3 — Six conserved motifs in C5-MTases. (A) Six conserved motifs of MET group, (B) Six conserved motifs of CMT group, and (C) Six conserved motifs of DRM2 groups. [file Image3.TIF]

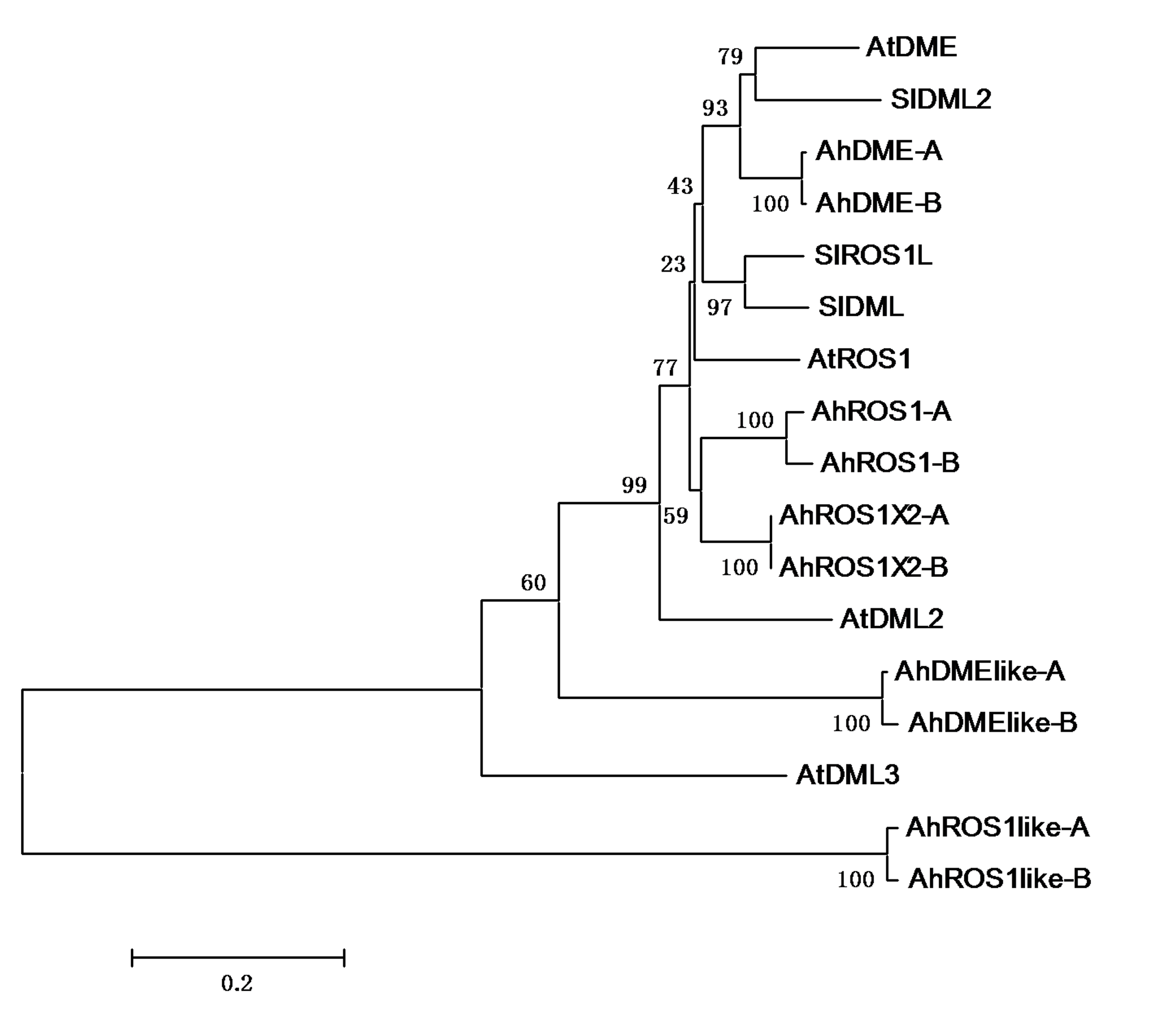

Supplement: Figure S4 — Neighbor-joining phylogeny of demethylases. [file Image4.TIF]

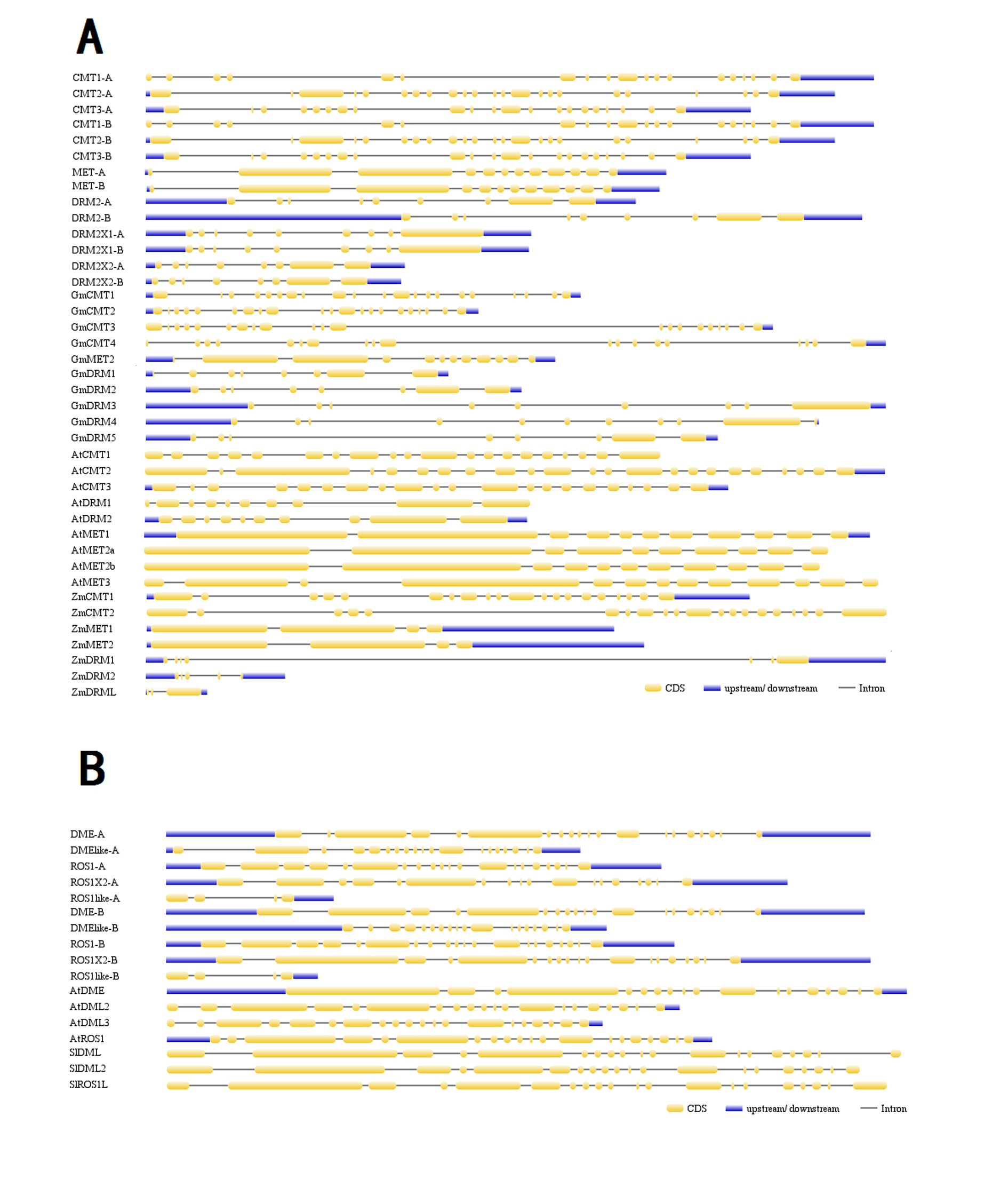

Supplement: Figure S5 — Gene structure of peanut C5-MTases and demethylases. (A) Gene structure of C5-MTases and (B) Gene structure of demethylases. [file Image5.TIF]

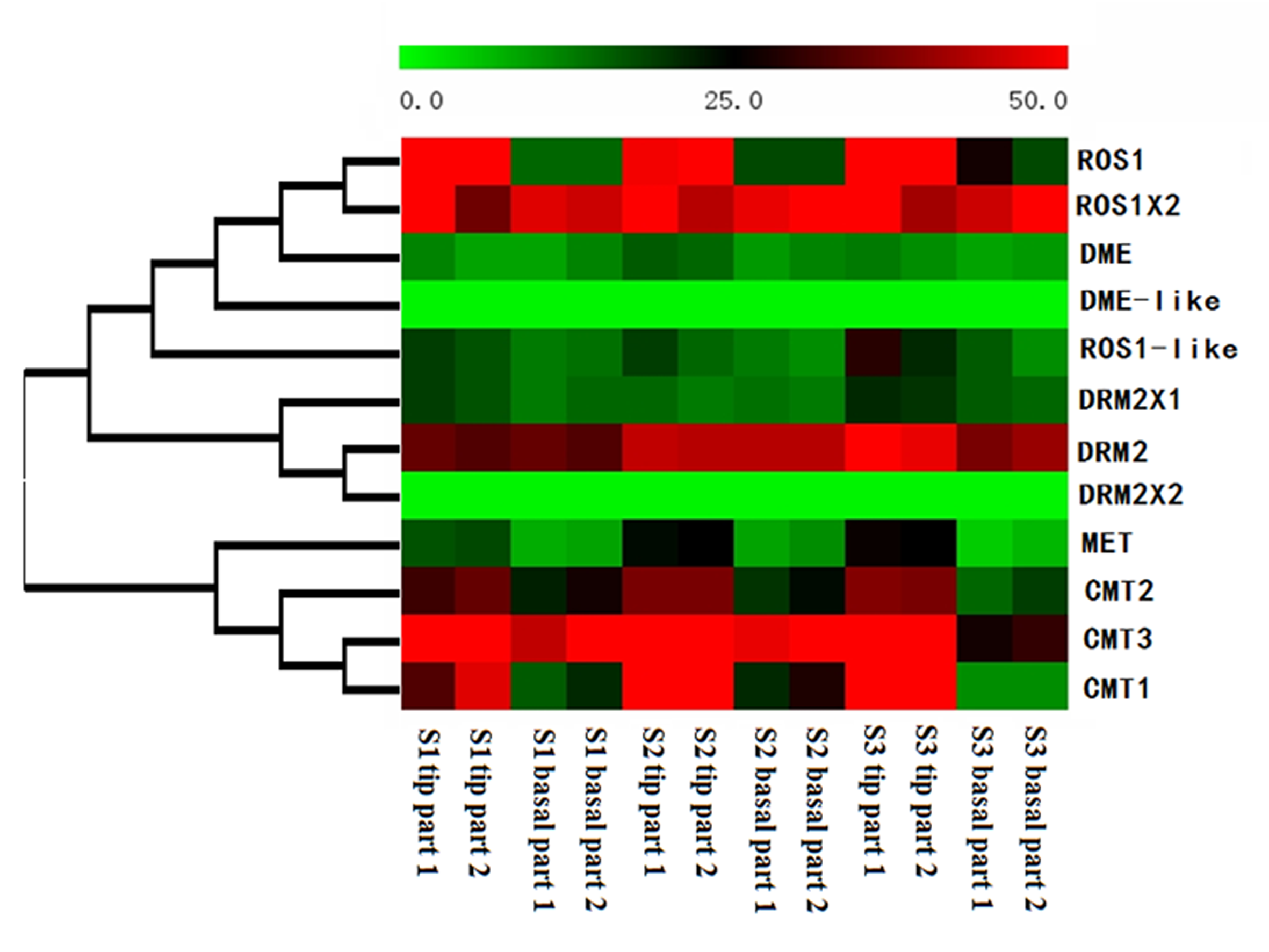

Supplement: Figure S6 — Heat map (using RPKM value) of C5-MTase and demethylase genes in different cultivated peanut tissue. [file Image6.TIF]

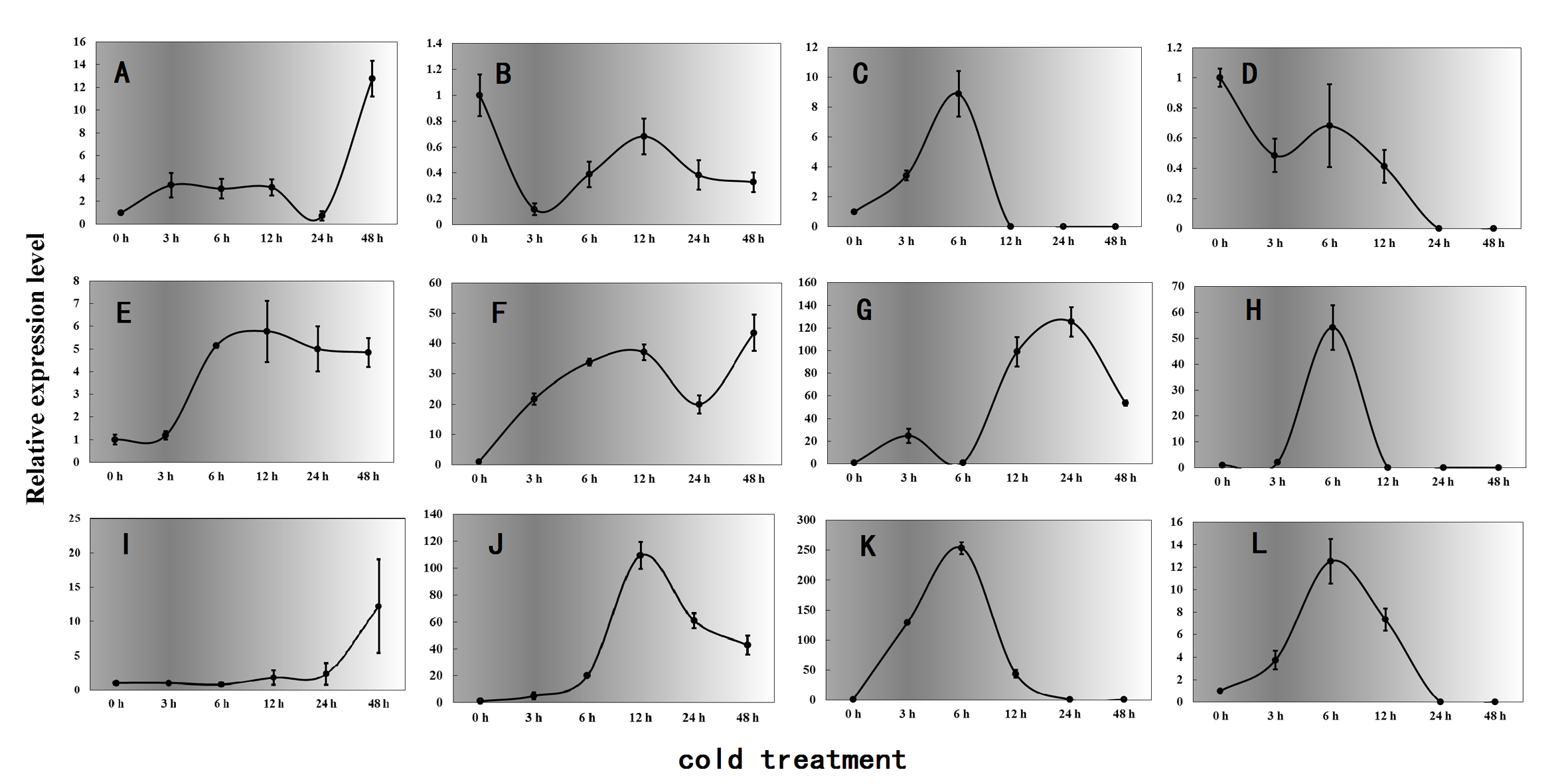

Supplement: Figure S7 — Relative expression levels of cultivated peanut C5-MTase and demethylase genes under cold stress. (A–L) respectively represent the relative expression levels of MET, CMT1, CMT2, CMT3, DRM2, DRM2X1, DRM2X2, DME, DME-LIKE, ROS1, ROS2-like, and ROS1X2 under cold stress from 0 to 48 h.“Y” axis represents relative expression level and “X” axis represents treated time (hour, h). [file Image7.TIF]

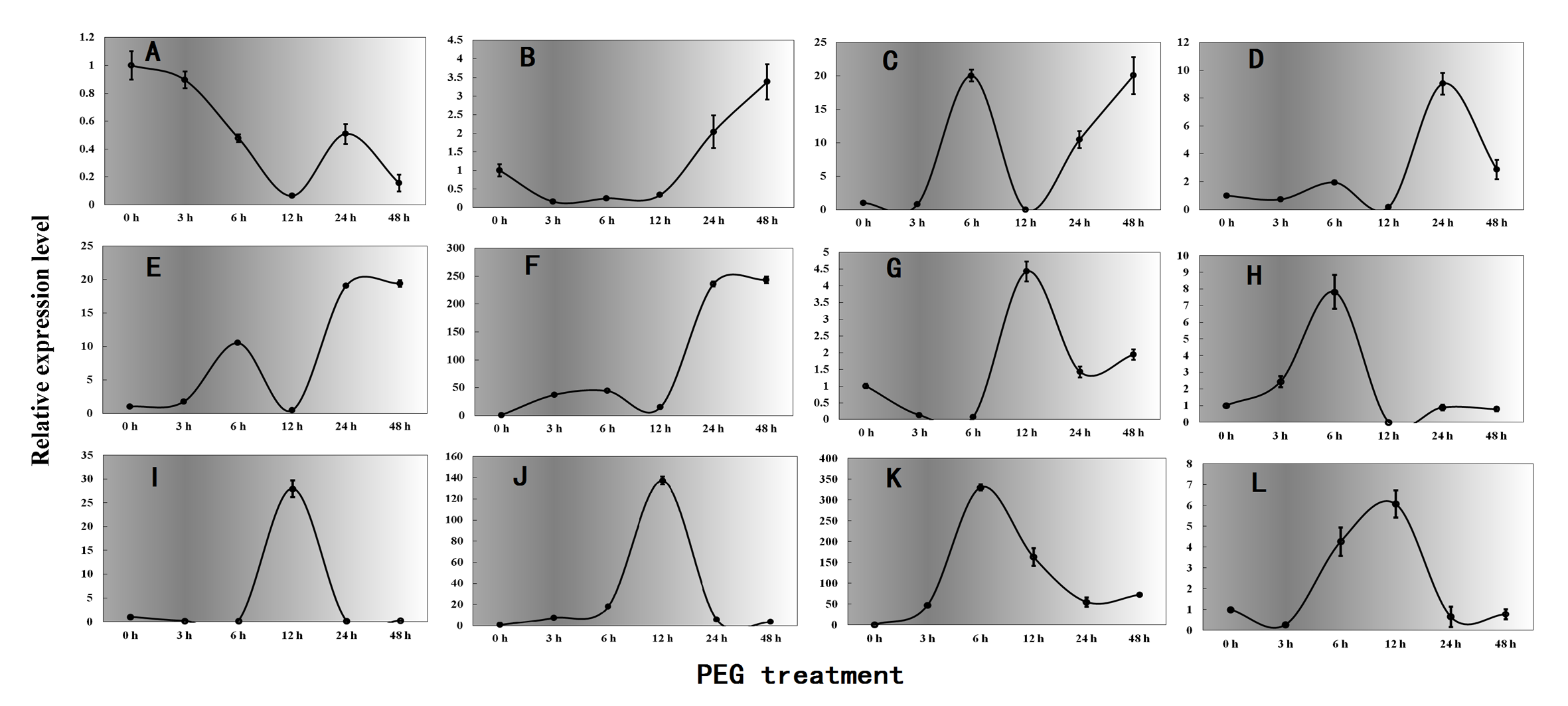

Supplement: Figure S8 — Relative expression levels of cultivated peanut C5-MTase and demethylase genes under PEG stress. (A–L) respectively represent the relative expression levels of MET, CMT1, CMT2, CMT3, DRM2, DRM2X1, DRM2X2, DME, DME-like, ROS1, ROS2-like, and ROS1X2 under PEG treatment from 0 to 48 h. “Y” axis represents relative expression level and “X” axis represents treated time (hour, h). [file Image8.TIF]

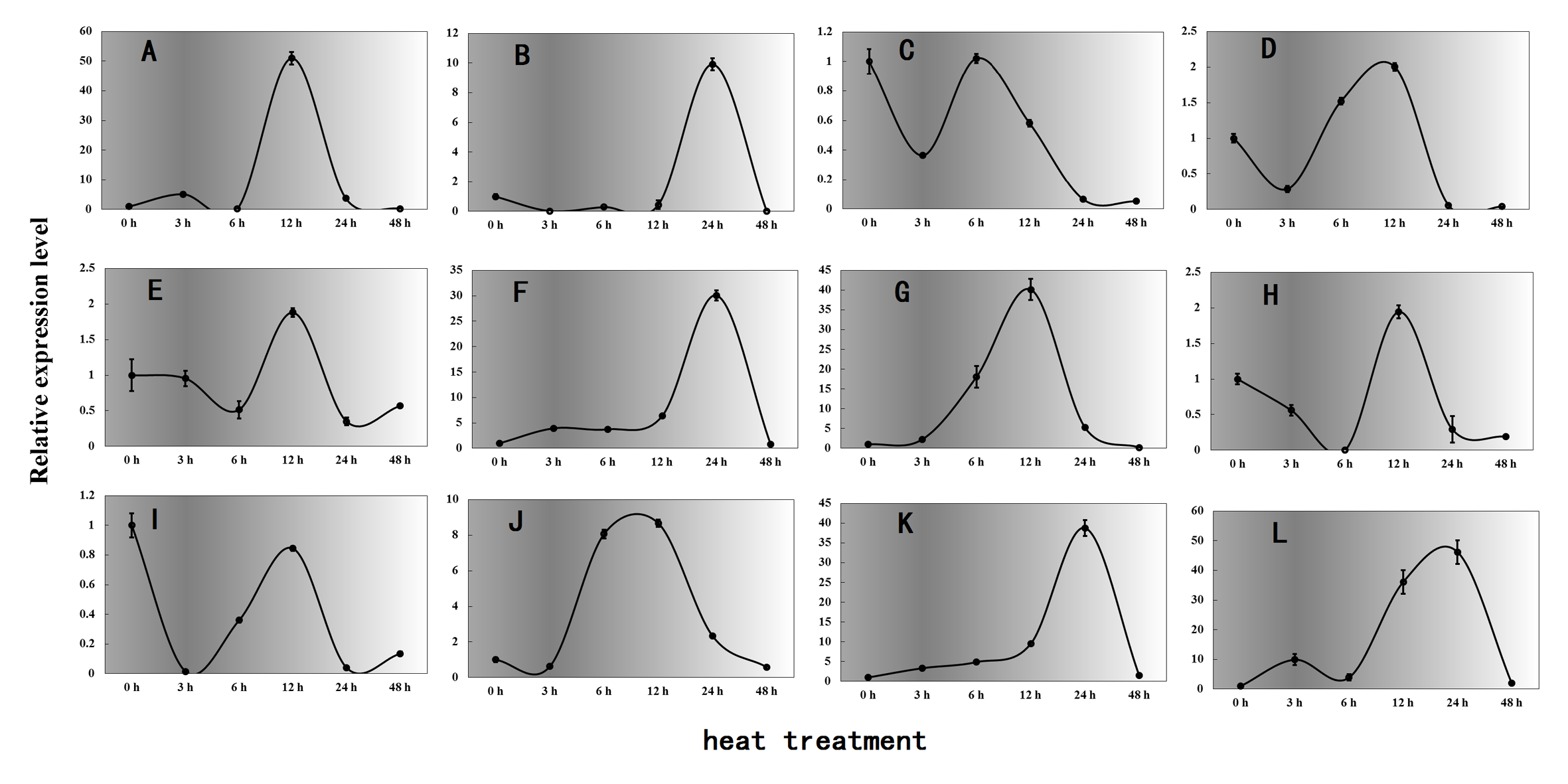

Supplement: Figure S9 — Relative expression levels of cultivated peanut C5-MTase and demethylase genes under heat stress. (A–L) respectively represent the relative expression levels of MET, CMT1, CMT2, CMT3, DRM2, DRM2X1, DRM2X2, DME, DME-like, ROS1, ROS2-like, and ROS1X2 under heat treatment from 0 to 48 h. “Y” axis represents relative expression level and “X” axis represents treated time (hour, h). [file Image9.TIF]
